# Tetraploidy in normal tissues and diseases: mechanisms and consequences

**DOI:** 10.1007/s00412-025-00829-1

**Published:** 2025-03-21

**Authors:** Micheline Kirsch-Volders, Miroslav Mišík, Michael Fenech

**Affiliations:** 1 Laboratory for Cell Genetics, Department Biology, Faculty of Sciences and Bio-Engineering Sciences, Vrije Universiteit Brussel, Pleinlaan 2, 1050 Brussels, Belgium; 2 Center for Cancer Research, Medical University of Vienna, Borschkegasse 8a, 1090 Vienna, Austria; 3 Genome Health Foundation, North Brighton, SA 5048 Australia

**Keywords:** Tetraploidy, Physiological tetraploidy, Unphysiological tetraploidy, Cardiovascular, Neurodegenerative, Diabetes

## Abstract

Tetraploidisation plays a crucial role in evolution, development, stress adaptation, and disease, but its beneficial or pathological effects in different tissues remain unclear. This study aims to compare physiological and unphysiological tetraploidy in eight steps: 1) mechanisms of diploidy-to-tetraploidy transition, 2) induction and elimination of unphysiological tetraploidy, 3) tetraploid cell characteristics, 4) stress-induced unphysiological tetraploidy, 5) comparison of physiological vs. unphysiological tetraploidy, 6) consequences of unphysiological stress-induced tetraploidy, 7) nutritional or pharmacological prevention strategies of tetraploidisation, and 8) knowledge gaps and future perspectives. Unphysiological tetraploidy is an adaptive stress response at a given threshold, often involving mitotic slippage. If tetraploid cells evade elimination through apoptosis or immune surveillance, they may re-enter the cell cycle, causing genetic instability, micronuclei formation, aneuploidy, modification of the epigenome and the development of diseases. The potential contributions of unphysiological tetraploidy to neurodegenerative, cardiovascular and diabetes related diseases are summarized in schematic figures and contrasted with its role in cancer development. The mechanisms responsible for the transition from physiological to unphysiological tetraploidy and the tolerance to tetraploidisation in unphysiological tetraploidy are not fully understood. Understanding these mechanisms is of critical importance to allow the development of targeted nutritional and pharmacological prevention strategies and therapies.

## Introduction

As compared to the diploid status (2N) present in most of human somatic cells, tetraploidy (4N) corresponds to a doubling of the normal number of chromosomes. Tetraploid cells contain four sets of the chromosomes characteristic of the species. Gametes are haploid (N) and contain only one set of these chromosomes as a result of meiotic division.

The DNA content of diploid (2N) or tetraploid (4N) cells depends on the stage of the cell cycle at which they are studied. 2N or 4N cells have a 2C or 4C DNA amount in G0/G1 phase of the cell before DNA replication phase (S phase), and 4C or 8C DNA after S phase, respectively. This doubling of DNA content is paralleled by the number of chromatids present in the chromosome: one chromatid per chromosome before S phase, two chromatids per chromosome after S phase. If more than 2 chromatids are present in a single chromosome it indicates that more than one S phase occurred without separation of the chromatids (endoreduplication).

During evolution, polyploidisation is one of the main routes to the growth of organismal complexity and evolutionary plasticity, and this occurs in all organism types, both in plants and animals. Polyploidy provides genomic and phenotypic plasticity that enables cells to evolve adaptive phenotype variants when they encounter new conditions. This plasticity is acting not only in evolution. It is conserved in somatic polyploidy where it fulfils developmental pathways and regeneration, including response to stress, DNA damage and hypoxia. But it may contribute also to aging and diseases, in particular cancer (for review (Anatskaya and Vinogradov 2022a, b)). It is presently considered that regeneration and carcinogenesis have similarities with evolution where the appearance of lower-ploidy cells from the amitotic division of polyploid cells is considered as one of the mechanisms of the origin of multicellular organisms. Indeed, regeneration and carcinogenesis may include the ability of certain types of multinucleated polyploid cells to enhance stem properties (for review (Anatskaya and Vinogradov 2022a, b))**.** These considerations converge to the conclusion that tetraploidisation/polyploidisation is a fundamental phenomenon in evolution, development, adaptation to stress and changing environments, and in diseases. However, the reasons why tetraploidy/polyploidy is beneficial in some cell types and tissues, and pathological in others are largely unknown. Our aim is to compare both situations by extracting the main features related to human health in 8 steps: 1) mechanisms leading from diploidy to tetraploidy in physiological conditions, 2) induction of unphysiological tetraploidy and checkpoints for its elimination, 3) characteristics of the tetraploid cell, 4) stress-induced unphysiological tetraploidy, 5) comparison of physiological and unphysiological tetraploidy, 6) consequences of unphysiological tetraploidy, 7) nutritional or pharmacological prevention of tetraploidy and 8) knowledge gaps and future perspectives.

## Mechanisms leading from diploidy to tetraploidy in normal physiological conditions

During mitosis, different steps are involved in the separation and segregation of chromatids towards two genetically identical mononucleated daughter cells. Before considering how and why some cells physiologically progress towards tetraploidy, we will first review briefly these steps and, in particular, the aspects which might help understanding the induction of tetraploidy.

### Major steps in the separation and segregation of chromatids in diploid cells

#### Centriole duplication 

A mature centrosome typically comprises a pair of centrioles embedded in a protein matrix, the pericentriolar material. Centriole duplication is initiated in the G1-S-phase and is controlled through a centrosome-intrinsic blockade, in which duplication of the parent centriole is prevented as long as the parent and the newly synthesized procentriole remain in tight orthogonal association. The dissolution of this linkage, first step towards centriole duplication, is known as centriole disengagement; it requires the activity of the kinase PLK1 and the protease separase, which permits the reduplication of the parent centriole in the next cell cycle (for review Galofré et al. 2020; Hoffmann 2022)). In a second step, the polo-like kinase PLK4, a central regulator of centriole assembly, is recruited by Cep152 and Cep192 to the centrosome to form with two structural proteins, STIL (SCL/TAL-interrupting locus protein) and Sas-6 (spindle assembly abnormal protein 6), the core of centriole duplication (for review Hoffmann 2022; Shin et al. 2021)). In telophase, the two centrioles containing each two centrosomes separate, migrate and define the two poles of the mitotic cell in pro-metaphase. This activity has to be tightly regulated during the cell cycle. Supernumerary centrioles causes mitotic delays which stabilize p53 through activation of LATS2 kinase in the Hippo pathway, the PIDDsome pathway and others. The presence of excess microtubules nucleated by extra centrosomes induces increased Rac activation and therefore lower Rho activity; the latter is known to indirectly but potently suppress LATS1/2 activity in the Hippo pathway, resulting in inhibition of YAP and induction of cell-cycle arrest (Ganem et al. 2014).

The PIDDosome, an activating platform containing caspase-2, specifically located at the mother centriole may be triggered by genotoxic stress (Janssens and Tinel 2012; Raff and Basto 2017) and supernumerary centrioles (for review Garcia-Carpio et al. 2023; Shin et al. 2021)). The activation of PIDDosome, leads to caspase-2-mediated MDM2 cleavage, p53 stabilization and p21-dependent cell cycle arrest (Burigotto et al. 2021; Evans et al. 2021; Fava et al. 2017)); for review (Garcia-Carpio et al. 2023; Shin et al. 2021)).

#### Attachment of chromosome kinetochores to the spindle fibers, sister chromatid separation and its control

Within a chromosome, sister chromatids are linked by a chromosomal protein complex, cohesin, that connects sister chromatids from the time they are generated in S phase onwards, and which is dismantled at the onset of anaphase through cleavage by the cysteine protease separase. Separase is inhibited prior to metaphase by the tightly bound securin protein (for review Rosen et al. 2019; Uhlmann 2003)).

In (pro)-metaphase, chromosomes attach to the spindle microtubules through the kinetochores, which are assembled onto the centromeric chromatin. The kinetochores also provide the signaling required to create communication between the state of microtubule attachment and the spindle assembly checkpoint (SAC) activity safeguarding faithful chromosome segregation. The SAC delays anaphase onset by generating a diffusible inhibitor known as the mitotic checkpoint complex (MCC), comprised of two subcomplexes, BubR1:Bub3 and Cdc20:Mad2. Once formed, the MCC binds and inhibits the anaphase-promoting complex/cyclosome (APC/C), an E3 ubiquitin ligase, whose ubiquitination activity is required to target cyclin B and securin for degradation, allowing cohesin cleavage by the separase (for review (Fischer 2023; Lara-Gonzalez et al. 2021)). Moreover CdK (cyclin-dependent kinase) inhibition and activation of Cdk-counteracting phosphatases are required to reverse the many phosphorylation events and allow mitotic exit (Kuilman et al. 2015).

#### Karyokinesis: from metaphase to anaphase and telophase

In metaphase, the sister chromatids are still linked together at the kinetochore-microtubule interface and maintained at the metaphase equatorial plate by the spindle, a self-organized structure, consisting of dynamic microtubules. In anaphase, microtubules are drawn apart towards the opposite poles of the cell. A bipolar spindle first pulls apart the sister chromatids, then a central spindle further separates them away. Aurora-A is a centrosome localised member of the Aurora kinases family. Aurora-A is involved in the centrosome maturation process and contributes to the activation of the PLK1 kinase, thus promoting the transition from G2 to mitosis. It regulates the assembly of the mitotic spindle from both centrosomes and chromosomes. Besides its role during mitosis, a non-mitotic function of Aurora-A was described in association with a nuclear localisation and potential functions in cancer (Naso et al. 2021).

In all eukaryotes, anaphase movement is driven by forces generated at three key sites: the kinetochores, the astral region around the centrioles, and the central spindle. The spindle microtubules can rapidly grow and shrink; molecular motors cross-link microtubules and induce their relative sliding. It is considered that the kinetochores transduce to the chromatids the forces from the dynamic microtubules. However, the mechanisms which ensure chromosome segregation are still unclear (for review (Anjur-Dietrich et al. 2021)).

During telophase, when the chromosomes reach the opposite poles, the nuclear membrane reassembles around each set of chromosomes, and the spindle fibers are dismantled and disappear, completing karyokinesis.

#### Cytokinesis

Cytokinesis corresponds to the division of the cytoplasm of the parent cell between the two daughter cells (for review (Fraschini 2020)). Several molecules participate in cytokinesis: the plasma membrane, filamentous actin (F-actin), type II myosin, tubulin, kinesin, dynein and anillin (for review Atilla-Gokcumen et al. 2010; Oliferenko et al. 2009; Piekny and Maddox 2010)). Cytokinesis starts (early telophase) with the development of the cleavage furrow along a plane that bisects the region between the segregated chromosomes. Next, furrow ingression occurs through contraction of an actomyosin ring that causes shrinkage of the ring circumference, midbody formation, drawing in the plasma membrane and progressively closing off the cytoplasmic connection between the two daughter cells. Finally, abscission takes place via membrane fusion (for review Lacroix and Maddox 2012; Oliferenko et al. 2009)). A coordinated interaction of several regulatory proteins is required for faithful separation of both nuclear and cytoplasmic contents (Lacroix and Maddox 2012). The small GTPase RhoA plays a central role in furrow ingression and its effectors activate ESCRT involved in abscission (for review (Koh et al. 2022)). Moreover, Bai et al. found that actin reduction by MsrB2 is a key component of the cytokinetic abscission checkpoint and prevents tetraploidy (Bai et al. 2020).

### From diploidy to physiological tetraploidy (or polyploidy)

Several mammalian cell types, such as hepatocytes, cardiomyocytes, megakaryocytes, cardiac interstitial and pericardial cells, and trophoblast giant cells, undergo tetraploidization and subsequent polyploidization. Additionally, multinucleated polyploid cells, which are characteristic of skeletal muscle cells, trophoblast giant cells, and osteoclasts, have been identified in the human kidney cortex. (Fantone et al. 2022), the urothelial barrier (Wang et al. 2018) and others such as skin, brain, breast and lung (for review (Zemans 2022)).

#### Mechanisms and examples

**Fig. 1:**
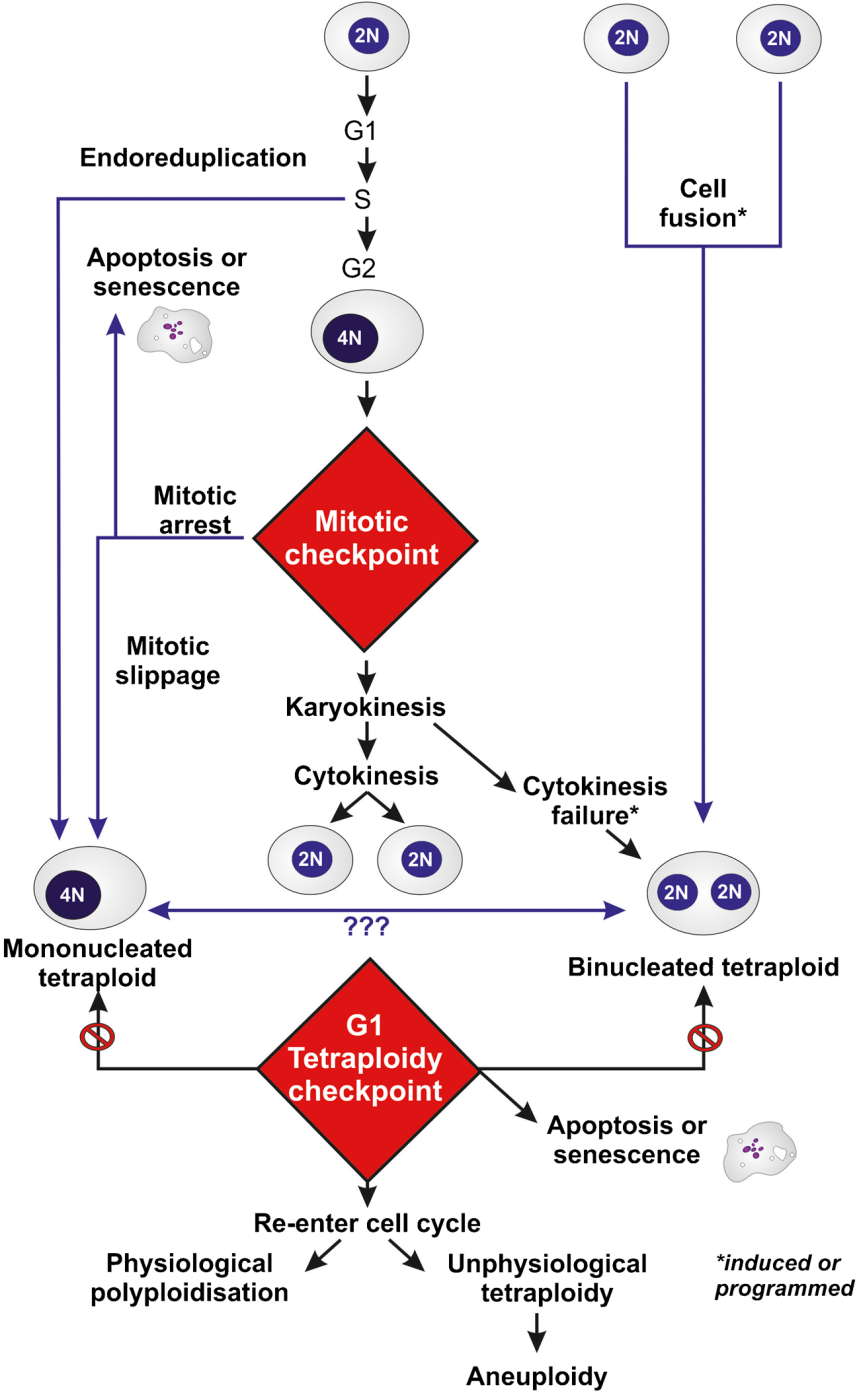
Schematic representation of the mechanisms leading to physiological and unphysiological tetraploidy. The “???” reflects the fact that the link is not fully demonstrated

In tissues that naturally evolve toward tetraploidy and, eventually, polyploidy, specific cells do not exhibit known deficiencies in the mechanisms controlling karyokinesis and cytokinesis. Instead, they progress toward tetraploidy and/or polyploidy through a programmed process, primarily driven by endoreduplication—a specialized cell cycle in which DNA replication occurs without subsequent karyokinesis or cytokinesis. Additionally, this progression is facilitated by the regulated inhibition of cytokinesis following normal karyokinesis or through cell fusion. (for review Peterson and Fox 2021; Zhang et al. 2020a, b)). Endoreduplication is frequent in plants and some animals but rare in mammals (for review (Larkins et al. 2001)). Figure 1 summarizes the different mechanisms leading to physiological and unphysiological tetraploidy.

##### Endoreduplication

Endoreplication also named endocycling, consists of repetitive G1/S-phases sequences (without G2, karyokinesis or cytokinesis) leading to amplification of chromatids, increased nuclear size and DNA content within a single nucleus, e.g. megakaryocytes. Megakaryocytes are the mother cells of blood platelets. They develop from diploid, bone marrow hematopoietic stem cells, which at a given stage replicate their genome, skip nuclear (karyokinesis) and cellular (cytokinesis) division, leading to tetraploidy and further to high levels of polyploidy (Machlus and Italiano Jr 2013). The regulation of megakaryocytes formation is still unclear.

##### Regulated inhibition of cytokinesis

Inhibition of cytokinesis results in the formation of binucleated (2 × 2N) cells, as seen in hepatocytes, cardiomyocytes, and urothelial barrier cells. Hepatocytes become tetraploid, and further on may become polyploid, as a result of incomplete cytokinesis, generating binucleated cells (2X2N) which function without cell division, and may become mononucleated (4N) after nuclear fusion. These 4N mononucleated tetraploid cells may undergo a new cycle and generate 2 mononucleated tetraploid cells (1 × 4N; 1X4N) or a binucleated octoploid cells (2X4N), if cytokinesis is inhibited again. Following this initial phase, hepatocyte polyploidy only increases modestly over the organism’s lifespan (for review (Sladky et al. 2021)). Up to 40% of hepatocytes in the adult human liver are polyploid, whereas this proportion is nearly twice as high in rodents.

Several pathways have been implicated in hepatic ploidy control. The main mechanisms regulating polyploidisation in liver are linked to insulin signaling in response to nutritional changes and transcriptional control of cytokinesis effector genes (E2F family, p53-p21 axis, Mkp1), but post-transcriptional effects involving microRNA (miR) action on cytokinesis factors such as RhoA and CUX1 and direct interference with the process of cytokinesis have also been reported (for review (Sladky et al. 2021)). Interestingly, the centrosome-PIDDosome-P53 signaling axis which is a part of E2F transcriptional regulatory circuit acts as a brake on hyperploidisation: the extra-centrosomes accumulated in polyploid hepatocytes activate the PIDDosome, leading to p53 stabilization and cell cycle arrest. These different pathways interact in complex ways that are not yet fully understood. While most of the data were obtained in rodent cells; recently interesting results were obtained on human hepatocyte organoids (Darmasaputra et al. 2024). Despite the differences in the mechanisms by which cells inhibit division, the authors uncovered a conserved function of WNT signaling along with E2F7 and E2F8 in the regulation of endomitosis, suggesting that similar mechanisms control the choice between canonical and non-canonical cell cycles in both rodents and human hepatocytes. Further research is required to provide perspectives for clinical implications in liver disease and hepatocellular carcinoma (for review (Donne et al. 2021; Sladky et al. 2021)).

Cardiomyocytes ploidy can range from diploid (2N) to 4N, 8N or even 16N. Nuclear polyploidy and multinucleation can be linked to different developmental stages and the changing regenerative capacity of the heart but the mechanisms underlying these ploidy changes are only currently being elucidated (Derks and Bergmann 2020). Several processes have been described through which they can become polyploid. Mononucleated polyploid cardiomyocytes can arise via karyokinesis failure leading to endocycling; cardiomyocytes can also become polyploid by multinucleation as a result of cytokinesis failure, in particular the abscission phase (for review (Kirillova et al. 2021)).

The majority of mammalian species assessed for cardiomyocytes ploidy display predominantly bi-nucleated cardiomyocytes but human cardiomyocytes are primarily mononuclear (75%) with increased nuclear polyploidy (for review (Buddell et al. 2024; Swift et al. 2023)). It was believed that due to their multiple or unpaired centrioles, multinucleated cardiomyocytes could not form bipolar spindle, resulting in cytokinesis failure. Other studies have shown that binucleated cardiomyocytes retain their capacity to proliferate at least in vitro. However, mononucleated cardiomyocytes enter into the cell cycle more frequently than polyploid cardiomyocytes (for review (Derks and Bergmann 2020)). Thyroid hormones might act as master regulators of metabolic changes, leading to either cardiomyocyte ploidy or proliferation (for review (Derks and Bergmann 2020)). Buddell et al. reviewed the genes involved in the regulation of cardiomyocyte ploidy. These genes encode cell cycle regulators, transcription factors, metabolic proteins, nuclear scaffolding, and components of the sarcomeres, among others (for review (Buddell et al. 2024)). As examples, several gene products such as decrease in Ect2 and Gas2/3, and increase in cyclin D1, cyclin G1 and GSK-3alpha/beta are found to regulate induction of multinucleation in cardiomyocytes. On the other hand, increase in YAP and decrease in cyclin G1 and TNNl3k stimulate cytokinesis. Karyokinesis failure leading to formation of mononucleated polyploid cardiomyocytes is regulated via the SMAD pathway and linked to a decreased level of lamin B2 (for review Buddell et al. 2024; Derks and Bergmann 2020; Kirillova et al. 2021)).

The urothelium is an epithelia barrier lined by a luminal layer of binucleated, octoploid, superficial cells. The superficial cells are likely to be derived from a population of binucleated intermediate cells, which are produced from mononucleated intermediate cells via incomplete cytokinesis. These binucleated cells further increase their DNA content, via endoreduplication, passing through S-phase without entering mitosis (Wang et al. 2018).

##### Cell fusion

Cell fusion leading to multinucleation (polyploidy) with many 2N nuclei within a syncitium, e.g. skeletal muscle cells, placental syncytiotrophoblasts and osteoblasts. Cell fusion involves several steps, beginning with cell–cell adhesion, followed by destabilization of the membrane lipid bilayer, and fusion of the two cells. It is a normal programmed step in the development of skeletal muscle cells, placental syncitiotrophoblasts and bone osteoclasts.

A skeletal muscle cell contains hundreds to thousands of nuclei within a shared cytoplasm. These fusion events are highly regulated with positive and negative factors to fine-tune the process in function of muscle development, regeneration and adaptations. Muscle-specific fusogens (Myomaker and Myomerger) as well as general cellular machinery to achieve the union of membranes are required ((Zhang et al. 2020a, b); for review, (Millay 2022)).

Placental syncytiotrophoblasts form the interface between the maternal blood and embryonic fluid. They are multinucleated, formed and maintained through fusion of the underlying diploid cytotrophoblast cells (Knöfler et al. 2019). The regulation of placental syncytiotrophoblast formation is still unclear.

Bone osteoclasts form through the fusion of mononucleated progenitors from the monocyte/macrophage lineage. Their multinucleation not only increases cell size but causes functional alterations through reconstruction of the cytoskeleton, creating the actin ring and ruffled border that enable bone resorption (for review Kodama and Kaito 2020; Søe 2020)). Multinucleation of osteoclasts is induced by DC-STAMP and OC-STAMP as “master fusogens”, including the La protein and others (for review Kodama and Kaito 2020; Whitlock et al. 2023)). However, how osteoclasts regulate their fusion and arrive at the adequate size to fulfill their function remains unclear.

#### Importance, advantages and disadvantages of physiological tetraploidisation/polyploidisation

In general, the presence of multiple identical genomes in tetraploid/polyploid mononucleated cells (nuclear polyploidy) is considered to provide opportunities for higher metabolic activity (several identical genomes transcribed simultaneously) and better protection against genotoxic stress (in case one gene copy is mutated, multiple gene copies remain intact). In the case of multinucleated polyploid cells, multinucleation favors synchronisation of transcriptional activity, localized high activity on the target(s) and cell shapes which facilitate their function (e.g. large polyhedric hepatocytes lining the liver canals, long shaped skeletal muscle cells, thin extended barrier of cells for human placental syncytiotrophoblast layer). However, long shaped cells can be very efficient as mononucleated diploid cell (e.g. neurons) and it is not clear why some tissues develop using mononucleated polyploid cells, multinucleated cells or a mixture of the two (for review, (Peterson and Fox 2021)).

A major disadvantage of polyploidy might be a lower proliferation and therefore regeneration capacity; however, it can be restored by reduction of polyploidisation through division of a binucleated tetraploid cell (2X2N) into two mononucleated diploid cells (2N and 2N) or re-entrance in the cell cycle of multinucleated cells followed by cytokinesis. Polyploidy presents additional disadvantages, such as no restoration of normal cell number after injury, predisposition to chromosome malsegregation if they do divide and tendency to lose replication ability and become senescent (for review (Zemans 2022)).

The high heterogeneity of cell types which undergo tetraploidisation, and further polyploidisation, suggests that it might have also specific physiological benefits. For hepatocytes, it is clear that the increased number of genomes provide enhanced gene transcription activity and protein synthesis, leading to hypermetabolic conditions (for review (Donne et al. 2021; Sladky et al. 2021)). For cardiomyocytes, numerous explanations for polyploidisation have been proposed, including protection against genotoxic stress and promotion of hypertrophy through inhibition of cell proliferation. Nonetheless, the physiological role of increased ploidy levels in these cells remains unclear. For osteoclasts, transcriptional activity of nuclei in multinucleated cells is modulated by calcitonin and shown to be the same for all the nuclei within a cell (Boissy et al. 2002; Derks and Bergmann 2020).

An interesting specific aspect of polyploidisation is its role in regeneration and repair. As an example, during lung regeneration polyploidy offers several advantages over cell proliferation as a means to restore tissue mass after injury, maintaining barrier integrity, and avoiding disassembly of cell–cell junctions and of cell–matrix junctions. It also protects against loss of tumor suppressors and against genotoxic stress/cell death. It is a reservoir for new diploid cells (for review (Zemans 2022)). Whether polyploidisation is also influencing the differentiation process is not clear. One may consider that polyploidization both in hepatocytes and cardiomyocytes is part of the normal differentiation process; on the other hand aneuploidy-derived-polyploidization may influence de-differentiation.

When trying to find a common characteristic to the different tetraploid/polyploid situations, providing plasticity for development, differentiation, repair, response to environmental stress or pathological conditions seems to be a major driver. Tetraploidy/polyploidy as such might not be sufficient to provide enough possibilities. Epigenetic modifications are considered as another, *in casu* additional, mediator of plasticity, leading to phenotypic plasticity (Anatskaya and Vinogradov 2022a, b; Feinberg and Levchenko 2023).

## Induction and checkpoints of (un)-physiological tetraploidy

### Inducers of unphysiological tetraploidy

From the mechanisms described above, it seems logical to consider that the most critical steps whose physiological modification or stress-induced deficiency may lead to tetraploidy, these are: kinetochore assembly at centromeres (for review (Dong and Li 2022)), kinetochore-microtubule attachment (for review (Dong and Li 2022)), chromatid separation at anaphase (Chu et al. 2022; Uhlmann et al. 1999), spindle microtubule activity (for review (Lakshmi et al. 2018)), centriole function (for review (Conduit et al. 2015)), and cytokinesis (for review (Lacroix and Maddox 2012)). Defects in these processes may result from gene mutations in key genes, from DNA damage-induced chromosome recombination, epigenetic silencing of critical genes from abnormal interactions with gene products essential for the mitotic machinery or from exposure to some viruses.

#### Gene mutations or overexpression

Several oncogene mutations were shown to induce whole genome doubling through mitotic slippage or suppression of cytokinesis. For example, BRAF mutation in melanomas increases the RAS/MAPK-pathway activity and causes in G1/S inappropriate centriole amplification which is linked in part to the inhibition of the small GTAPase RhoA, a critical cytokinesis regulator (Darp et al. 2022). It has been reported that stress-related overexpression of Myc drives polyploidy by promoting rapid S-phase entry, DNA replication, and mitotic spindle abnormalities. Conversely, polyploidy itself upregulates Myc through mechanisms involving stress responses, genetic instability, and replicative stress linked to chromatin opening. This creates a self-reinforcing cycle, perpetuating a circle of polyploidization and Myc activation (Anatskaya and Vinogradov 2022a, b).

As examples for mutations in tumor suppressor genes, one may mention loss of function of APC (adenomatous polyposis coli), BRCA1, BRAC2 and/or TP53 which induce polyploidy as a result of a combination of defects in mitosis and apoptosis. Loss of APC leads to a partial loss of interkinetochore tension and compromises the mitotic spindle checkpoint. It is accompanied by a decrease in the association of the checkpoint proteins Bub1 and BubR1 with the kinetochore (Austria et al. 2018; Dikovskaya et al. 2007).

Recently a review by Choudhury et al. suggests a molecular link between mTOR signaling and polyploidy (Choudhury et al. 2024). They found a strong correlation between the hallmarks of polyploidy and mTOR signaling, along with its association with cancer, aging and diabetes.

#### Induction of oxidative stress, DNA damage, chromosome recombinations and aneugenic stress

DNA damage and structural chromosome aberrations can result from DNA replication stress or exposure to physical (ionising radiation, UV) or chemical mutagens, including reactive oxygen and nitrogen species (RONS); also biological agents (viruses) may be mutagenic.

Oxidative stress, which is induced by intracellular metabolism or extracellular factors, can act as an inducer of physiological polyploidisation leading to differentiation, as well as an inducer of pathological polyploidy. In cardiomyocytes, the metabolic rate and the concurrent RONS production seem to be linked to their ploidy status. Indeed, there are indications that increased oxygen stress, such as after birth, is linked to DNA damage, subsequent cell cycle arrest and induction of binucleation. Accumulation of genomes during metabolic maturation coincides with RONS overproduction and genome instability due to lamina reorganization and transition to the oxygen-rich postnatal environment (for review (Anatskaya and Vinogradov 2022a, b)). Oxidative stress was also found to promote pathological polyploidisation in nonalcoholic fatty liver disease (Gentric et al. 2015).

Interestingly, hypoxia may also trigger polyploidisation. Various heart diseases can induce cardiomyocytes loss and therefore hypoxia. Hypoxia can induce polyploid and multinucleated cardiomyocytes by increasing factors related to the G1-S-anaphase of the cell cycle, but not those related to cytokinesis (Jiang et al. 2019). On the other hand, it was found that cytokinesis can be promoted through activation of beta-catenin (Derks and Bergmann 2020; Jiang et al. 2019) and that the multinucleated polyploid cardiomyocytes undergo an enhanced adaptability to hypoxia via mitophagy (Jiang et al. 2020).

Replication stress, if inducing DNA lesions in the S phase which are carried over into the M phase, can cause DNA cytokinesis failure and tetraploidy (Ichijima et al. 2010).

DNA damaging agents that induce DNA breakage and structural chromosome aberrations are named clastogens. If recombination of two chromosomes occurs after DNA breakage, a dicentric chromosome may be formed which may lead to anaphase bridge formation. The latter may hamper cytokinesis, induce cytokinesis failure and lead to tetraploidisation. A particular type of chromosome aberrations resulting from telomere shortening and provoking telomere crisis, is associated with an increase in ploidy. Excessive telomere shortening may lead to telomere fusion, formation of dicentric chromosomes. These dicentric chromosomes may cause anaphase bridge formation and hinder the completion of cytokinesis which result in the formation of tetraploid cells (Davoli and de Lange 2012).

As far as aneugenic stress is concerned, it was shown, using cell culture systems, that perturbation of microtubule dynamics by either tubulin stabilizing or destabilizing drugs leads to sustained activation of the spindle assembly checkpoint (SAC), cell arrest in mitosis, mitotic slippage and tetraploidisation (Cheng and Crasta 2017). Similarly, perturbation of filaments involved in cytokinesis leads to cytokinesis failure and tetraploidisation (for review, (Lacroix and Maddox 2012)). Examples will be given in the following section, as illustrations of specific stress-related induction of tetraploidy.

#### Viruses

Enveloped viruses, which include common human pathogens and most of the known oncogenic viruses, enter cells with the help of viral proteins that fuse biological membranes, which as a consequence cause the fusion of cells. Some non-enveloped viruses also express proteins that fuse cells (for review (Duelli and Lazebnik 2007)). For example, the human papillomavirus (HPV) which is the causative agent of most cervical cancers, expresses oncogenes E7 and E6, deregulates the cell cycle, induces chromosome instability and prevents apoptosis, but is not sufficient to make cells cancerous (Cosper et al. 2023). A virus such as herpesvirus, which is a co-factor in induction of cervical cancer, is considered to fuse cells infected with HPV, producing tetraploid and polyploid cells observed in cervical malignant lesions (Heselmeyer et al. 1996). Infection by hepatitis B and C viruses, and the measle virus, also leads to cell fusion and polyploidisation (Chuprin et al. 2013; Duelli and Lazebnik 2007).

HIV is known to promote genome reduplication, through the Vpr protein. In human renal tubule epithelial cells, Vpr first initiates G2 cell cycle arrest. Next a fraction of cells re-enter the cell cycle and undergo mitotic cell death due to extra centrosomes and aberrant division; the others that abort mitosis undergo endoreduplication and become polyploid (Payne et al. 2018).

#### Tetraploidy induced by pathological conditions

Accumulation of genome copies in cells under pathological conditions are described in neurodegenerative disorders, cardiovascular diseases, diabetes, aging and wound healing (Anatskaya and Vinogradov 2022a, b; Choudhury et al. 2024).

In the nervous system, cells that are normally non-dividing or postmitotic can re-enter the cell cycle and begin replicating their DNA to facilitate cellular growth in response to cell loss. This may lead to (hyper)polyploidisation which may play a protective role against DNA damage or oxidative stress, but recent studies discovered a link between cell-cycle reentry in neurons, synaptic dysfunction and several neurodegenerative conditions. A possible explanation of this disparate associations is that cell cycle re-entry that proceeds into mitosis leads to neurodegeneration, while partial cell cycle re-entry is neuroprotective (for review (Nandakumar et al. 2021)).

In Alzheimer disease, it is known that the amyloid-beta peptide, the major component of senile plaques in the brain, triggers cell-cycle re-entry in fully differentiated neurons and that this cell cycle re-entry protects from amyloid-beta toxicity and resultant cell death (Ippati et al. 2021). Id1/HIF-1 and CdK5/HIF-1 were shown to contribute to amyloid-beta-induced cell cycle entry (Chao et al. 2020).

As far as cardiomyocytes are concerned, hypertrophy induced by hypertension leads to an increase in ploidy in human hearts; re-entering into mitosis of post-mitotic cardiomyocytes, followed by endomitosis and polyploidization, also occur during cardiac pathogenesis of diseases, such as ischemic and non-ischemic heart failure and arrhythmia (Meckert et al. 2005) (for review Buddell et al. 2024; Derks and Bergmann 2020)).

When investigating the development of polyploidy in pancreatic β-cells of normal and diabetic mice, the percentage of polyploid nuclei is significantly higher in diabetic mice compared to controls. Specifically, the proportion of tetraploid nuclei in diabetic animals is elevated by 220% over controls at 4.5 weeks of age, remains stable until 12 weeks, and then increases significantly between 12—14.5 weeks (Pohl and Swartz 1979). People with insulin-independent diabetes has a notably elevated proportion of polyploid nuclei in their islets. Individuals with insulin-dependent diabetes also have a much higher proportion of polyploid nuclei in their islets than the control subjects (for review (Choudhury et al. 2024)). However, recent in vitro studies showed that methylglyoxal (MGO), a reactive dicarbonyl by-product of glycolysis that is elevated in type 2 diabetes, causes mitotic dysfunction and complex nuclear anomalies, suggestive of mitotic slippage and aneuploidy, by preventing sister chromatid separation (Donnellan et al. 2021; Donnellan et al. 2022; Schalkwijk and Stehouwer 2020).

### Mechanisms leading to unphysiological tetraploidy

**Fig. 2:**
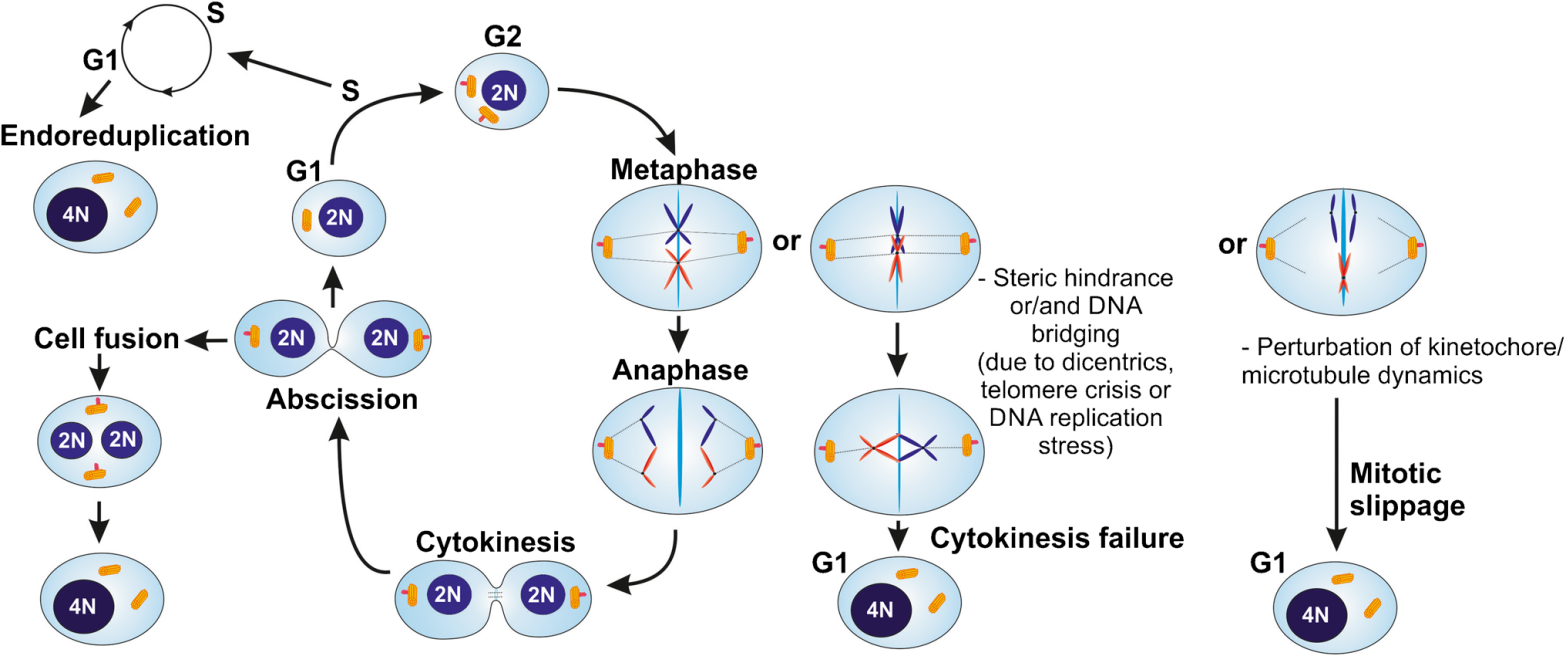
Comparison of the different mechanisms and actors involved in the induction of unphysiological tetraploidy, including centriole duplication, kinetochore attachment defects, spindle fibers, steric hindrance and DNA bridging (adapted from Lacroix and Maddox 2012)

The different possible mechanisms and actors leading to unphysiological tetraploidy are compared in Fig. 2.

#### Mitotic slippage leading to the formation of a mononucleated tetraploid cells

Depending on the biological context and cell type, cells blocked in mitosis can i) complete cell division, often leading to chromosomal mal-segregation and aneuploidy, ii) undergo mitotic cell death or iii) undergo mitotic slippage (Cheng and Crasta 2017; Gentric et al. 2015). Mitotic slippage is a phenomenon in which cells exit mitosis without properly completing chromosome segregation and cytokinesis, often leading to tetraploidy; it was described in vitro in human lymphocytes exposed to spindle poisons (e.g. nocodazole, tubulin depolymerising chemical) already in the late 90’s (Elhajouji et al. 1998).

The primary mechanism for mitotic slippage is the continued degradation of cyclin B1 (CCNB1) during the mitotic arrest, although SAC activity prevents fast degradation. The decrease of cyclin B1 below the threshold required to maintain the mitotic state, leads to mitotic exit (for review, (Nakayama and Inoue 2016)). In normal condition, cyclin dependent kinase-1 (CdK 1) forms a complex with cyclin B1 that phosphorylates substrates to drive cells into mitosis. This cyclin B1-CdK1 complex must be inactivated through the degradation of cyclin B1 allowing the cell to exit mitosis. The anaphase-promoting complex/cyclosome (APC/C), an ubiquitin ligase, known to polyubiquinate cyclin B1 to promote its degradation at the metaphase/anaphase transition is inhibited by the SAC until the chromosomes are properly aligned at the metaphase plate. In the absence of adequate microtubule fibers and no attachment of kinetochores on microtubules, cells arrest in mitosis, SAC is constitutively activated, and even in the absence of APC/C activity cyclin B1 degradation occurs (Brito and Rieder 2006). This is mediated by another ubiquitin ligase (CRL2), leading to mitotic slippage (Balachandran and Kipreos 2017). More recently Lok et al. found that mitotic slippage is determined by p31^comet^ and the weakening of the spindle-assembly checkpoint (Lok et al. 2020).

By mitotic slippage the cells escape cell death. The induction of apoptosis in mitotically arrested cells has been associated with accumulation of DNA damages during prolonged mitosis, activation of the p53 pathway and of the DNA damage repair response (for review (Ghelli Luserna di Rorà et al. 2019)). In their study, the authors propose a "competing networks-threshold" model, suggesting that during mitotic arrest, a cell's fate—whether undergoing mitotic slippage or apoptosis—is determined by the relative rates of cyclin B1 degradation and pro-apoptotic signal production (Ghelli Luserna di Rorà et al. 2019). If the decrease of cyclin B1 is slower than the increase of pro-apoptotic signals, the threshold favors mitotic death. On the contrary, if the degradation of cyclin B1 is more rapid than the production of pro-apoptotic signals, the threshold is in favor of mitotic slippage. In addition, other factors were found to modulate mitotic slippage: activation of the mitochondrial apoptotic pathway, activity of CRL2, dynamics of Mcl-1 (a member of the Bcl-2 family) and level of cyclin-dependent kinase 1 (CdK1) (for review (Cheng and Crasta 2017)).

#### Cytokinesis failure leading to the formation of a binucleated tetraploid cells

Cytokinesis failure may occur for different reasons (for review Kirsch-Volders and Fenech 2023; Lacroix and Maddox 2012)).

i) presence of DNA bridging across the anaphase spindle, due to errors in replication or condensation, or damage by mutagens or oxidative stress. Some cells with DNA bridges do perform cytokinesis and undergo apoptosis (Chu et al. 2002; Forrester et al. 1999; Tolmach 1990). But DNA bridges often inhibit cell separation by preventing abscission causing cell fusion and tetraploidy (Huang et al. 2008; Mullins and Biesele 1977); if the DNA damage is resolved in the next cell cycle, the cell may become stably tetraploid and avoid cell death.

ii) chromosome nondisjunction. If chromatin, a whole chromosome (or a chromosome bridge) is trapped between two dividing cells, it may induce cytokinesis failure; however it may be rescued by Aurora B kinase promoting proper chromosome segregation by delaying abscission (Pike et al. 2016; Steigemann et al. 2009) (for review Chen and Doxsey 2009; Zhao and Guan 2014)).

iii) telomere crisis (Zhao and Guan 2014). For example, in acute leukemia, it was shown that telomeres are particularly short in tetraploid/near-tetraploidy cells as compared to normal cells, suggesting that telomere dysfunction in hematological malignancies may be a mechanism for tetraploidization, possibly by causing telomere end-fusion causing dicentric chromosome formation leading to nucleoplasmic bridges which in turn could interfere with cytokinesis (Kjeldsen 2017).

iv) insufficient forces in the contractile ring to deform the cell due to inadequate constriction or activation of the ring. As an example, it was shown that actin filament poisons (e.g. dihydrocytochalasin B induce binucleated cells) (Carter 1967; Eastmond and Tucker 1989; Fenech and Morley 1986).

v) slow ring closure which can leave a cytoplasmic connection between the daughter cells and an out of time window for performing cytokinesis, leading to furrow regression (Canman et al. 2000).

vi) mechanical interference with contractile ring closure. As an example, it was shown that, in contrast to short multiwalled carbon nanotubes (approximately 5 µm), long multiwalled carbon nanotubes (approximately 20 µm) remained inside the contractile ring and induced the formation of binucleated cells through impairment of cytokinesis. This toxicity mechanism has also been observed with crocidolite, a kind type of asbestos fiber (Yasui et al. 2015).

vii) entosis, which describes the engulfment of a live cell by another (Krajcovic et al. 2011; Overholtzer et al. 2007). The host cell often fails cytokinesis because the internalized cell blocks furrow ingression (Lacroix and Maddox 2012).

#### Altered centrosome dynamics

When centrosomes are experimentally removed or disrupted, cytokinesis is impaired and cells arrest in the following G1 with tetraploid genomes. In addition, impairment of centrosome integrity activates a p38-p53-p21 checkpoint akin to the “G1 tetraploidy” checkpoint induced by mitotic slippage (for review (Aylon and Oren 2011)). More recently it was shown identified that polo-like kinase 4 (PLK-4) which regulates centriole duplication may trigger the induction of polyploidy and apoptotic death in case of deregulation (Kawakami et al. 2018); and that Eg5, a motor protein regulating centrosome separation, may lead to polyploidy through cytokinesis failure but not to post-slippage apoptosis in case of deregulation (Ohashi et al. 2015) (for review (Cheng and Crasta 2017)).

#### Cell fusion

Cell fusion may be caused by viral infection or by cell-to-cell fusion during malignant cell transformation (Duelli and Lazebnik 2007; Lu and Kang 2009). In addition, failure of timely membrane abscission can also lead to fusion of the daughter cells (for review (Lacroix and Maddox 2012)).

#### Chronic inflammation

Chronic inflammation, and thus chronic release of RONS, may lead to major health problems (for review (Shin et al. 2021). For instance, during wound healing, inflammation is the first step towards repair (for review (Eming et al. 2007)) and it was shown that polyploidisation by cell fusion is important for the replacement of cell loss (Losick et al. 2013). In response to persistent inflammatory stimulus such as mycobacterial disease (tuberculosis), vasculitis, inflammatory bowel disease, and sarcoidosis, formation of a granuloma is observed. At its core, the granuloma consists of different macrophages subsets with a range of morphologies, including binuclear and multinuclear cells (for review (Herrtwich et al. 2016)). It was generally believed that their polyploidisation can be explained by cell-to-cell fusion (Helming and Gordon 2009), but recent data demonstrated that TLR2 signaling promoted their polyploidisation via mitotic defect and not by cell fusion (Herrtwich et al. 2016). More recently it was shown that DNA damage signaling is an important trigger of polyploidisation of macrophages in chronic inflammation (Horn and Triantafyllopoulou 2018). Interestingly, Shi and King (2005) observed in human cell lines that spontaneous nondisjunction yields tetraploidy rather than aneuploid cells. However additional research is needed to understand the mechanisms responsible for induction of tetraploidy in cell lines. Although the different mechanisms involved in the induction and consequences of unphysiological tetraploidy are still unclear, one may consider that unphysiological tetraploidy is an adaptive defense mechanisms against endogenous and exogenous stressors.

### G1 tetraploidy checkpoint activation and immunosurveillance

The mechanisms governing tetraploidy-induced arrest in G1 are not well understood compared to cell death during mitotic arrest (for review Decordier et al. 2008; Nakayama and Inoue 2016; Zhao and Guan 2014)). Normally, cells possess robust intrinsic checkpoints, programmed cell death pathways (Vitale et al. 2011a, b) or immunological surveillance mechanisms preventing the survival of unphysiological tetraploidy (Senovilla et al. 2012)*.* However, the concept that some mechanisms may detect whether the number of chromosome sets is abnormal in unphysiological tetraploidy may seem unrealistic. The fate of tetraploid cells remains an important research topic, due to their role in cancer progression and importance as targets for chemotherapy and immunotherapy.

#### The G1 tetraploidy checkpoint and elimination of tetraploid cells

Tetraploid cells have been shown to adopt different cell fates, including re-entering the cell cycle as polyploid cells leading to chromosomal instability, arrest at the G1 tetraploidy checkpoint, followed by senescence or cell death by apoptosis (for review (Cheng and Crasta 2017)). Some tetraploid cells may proceed to DNA replication and be arrested in G2, such as binucleated tetraploid cells produced by absence of substrate adhesion during cytokinesis which enter senescence after DNA replication (De Santis Puzzonia et al. 2016).

The G1 tetraploidy checkpoint is mediated by p53 and pRb. pRb can inhibit the G1/S transition by binding E2F, the main activator of the genes regulating this transition. The Hippo pathway as a major controller of the cell cycle may act in two ways, by preventing (MST1/2, SAV1, LATS1/2) or stimulating (YAP/TAZ) polyploidization at the G1 tetraploidy checkpoint (for review (Xiao and Dong 2021)). Some other proteins may be involved in tetraploid G1 arrest, such as the F-box protein Skp2 for actin damage-induced tetraploidy (Jo and Shin 2017). However, p53 is considered as the key effector of the G1 tetraploidy checkpoint (for review Aylon and Oren 2011; Davaadelger et al. 2014; Ho et al. 2010)) and the induction of apoptosis in tetraploid cells (Castedo et al. 2006). In case of prolonged mitosis p53 accumulates and in a way « measures» the duration of the mitosis (Blagosklonny 2006).

Tetraploidy resulting from mitotic slippage after treatment of primary human lymphocytes by nocodazole can induce apoptosis and was shown to be dependent on p53 (Verdoodt et al. 1999). However, by comparing two paired human transformed cell lines, respectively expressing or not p53, it was found that G1 arrest in tetraploid cells after exposure to nocodazole requires p53 function in the re-replication checkpoint which prevents the G1/S transition following aberrant mitosis. In contrast, in the same paired transformed cell lines, p53 was found dispensable for triggering the apoptotic response in the absence of mitotic spindle (Casenghi et al. 1999; Verdoodt et al. 1999). Andreassen et al. found that the tetraploid state, rather than failure of spindle function, induces p53-dependent arrest of non-transformed mammalian cells in G1 (Andreassen et al. 2001). More recently, Zhu et al. found that the DNA damage response pathway was primarily responsible for post-slippage apoptosis after exposure to paclitaxel in a p53-dependent manner (Zhu et al. 2014).

Besides apoptosis which corresponds to programmed cell death, mitotic slippage may induce cellular senescence. Senescent cells are irreversibly arrested from the cell cycle but remain viable and develop a pro-inflammatory senescence-associated secretory (SASP) phenotype (Kirsch-Volders and Fenech 2023). Few data are available. It was found that paclitaxel treatment enhances mitotic-slippage associated senescence in cells with reduced Mad2 or BubR1 levels; similar data are mentioned after exposure to nocodazole (for review, (Cheng and Crasta 2017)). Deng et al. found that BAX/BAK prevents the induction of tetraploidisation associated senescence program. Indeed, cells lacking BAX and BAK, known for their role in the induction of apoptosis, proliferate less than their controls (or either BAX or BAK) by activating a senescence program when driven into tetraploidy by transient exposure to nocodazole (Deng et al. 2021). Also, failure of cell cleavage was shown to induce senescence in tetraploidy primary cells (Panopoulos et al. 2014). As far as apoptosis is concerned, the members of the B-cell lymphoma 2 (Bcl-2) family are known to be the main regulators of the intrinsic apoptosis pathway; however, multiple emerging evidence has revealed that the Bcl-2 family proteins are also involved in cellular senescence activated after tetraploidization. Apoptosis is characterized by the upregulation of proapoptotic Bcl-2 proteins and the downregulation of antiapoptotic Bcl-2 proteins. In contrast, senescence is typically associated with an increase in antiapoptotic Bcl-2 proteins and a decrease in proapoptotic Bcl-2 proteins (for review, (Barriuso et al. 2023)). However, why and when cells after mitotic slippage evolve towards senescence or apoptosis is not clear (for review (Kirsch-Volders and Fenech 2023)). Considering its critical impact on aging and cancer, this question will require innovative studies integrating high throughput specific markers and single cell approaches (Kirsch-Volders and Fenech 2023).

Cells that fail to undergo cytokinesis are also considered to activate a “G1 tetraploidy checkpoint” that arrests them in the following G1 in a p53-dependent manner. However, some studies suggested that this arrest does not occur because cells are polyploid, are binuclear, have multiple centrosomes or have failed cytokinesis (for review (Stukenberg 2004)); however, on the contrary, other studies considered that some mammalian cells may lack checkpoints for tetraploidy, aberrant centrosome number, and cytokinesis failure (Uetake and Sluder 2004; Wong and Stearns 2005).

The impact of centrosome number on the fate of tetraploid cells is gaining interest. Tetraploid cells harbour extra-centrosomes which may hamper the normal mitotic process. In general, the cells cycle further but show an increased occurrence of lagging chromosomes leading to aneuploidy (for review (Aylon and Oren 2011)). Whether p53 is involved or not in centrosome control has previously been debated (Borel et al. 2002). Interesting data indicate that extra centrosome-induced activation of the Hippo-pathway kinase LATS2 maybe a key mechanism of tetraploidy-induced cell-cycle arrest. However important questions remain unsolved: inhibition of LATS2 by Rho, mechanisms of LATS2 in p53 and cell cycle regulation (Zhao and Guan 2014).

#### Immunological surveillance

Tetraploid cells can be eliminated by the immune system. Interesting in vivo experiments demonstrated that the subcutaneous injection of tetraploid *TP53* ^−/−^ colon organoids led to the generation of proliferating tumors in immunological deficient but not in immunocompetent mice (Boileve et al. 2013). The same group found that hyperploid cancer cells become immunogenic because of a constitutive endoplasmic reticulum stress response resulting in the aberrant cell surface of calreticulin; injection of these hyperdiploid calreticulin-exposing cancers cells proliferate readily in immunodeficient mice and only after a delay in immunocompetent mice (Senovilla et al. 2012). Similar results were obtained after injection of fibrosarcomas cells induced in vivo in mice by methycholanthrene and, in particular, if the tetraploid fibrosarcomas cells were derived from immunodeficient mice (Bloy et al. 2015). More recently, they investigated the role of different innate and acquired immune effectors by inoculating hyperploid cancer cells (obtained by cloning of in vitro nocodazole-treated MCA and ELU cells) into wild type or mice bearing different immunodeficient genotypes, followed by the monitoring of tumor incidence, growth and final ploidy status. Their results suggest that multiple different immune effectors including B, NK, NKT and T cells, as well as innate immune responses involving the interleukine-1 receptor and the Toll-like receptor systems participate to the immunoselection against hyperploid cells (Aranda et al. 2018). This suggests that immunosurveillance is likely involving the contribution of multiple arms of the immune system.

### Tolerance to unphysiological tetraploid cells

Some genetic background seems to be able to favor survival and proliferation of tetraploidy cells. In 2015, Pan et al. observed that FLJ25439, a cytokinesis-associated protein, induces and stabilizes tetraploidy via enhancing expression of endoplasmic reticulum stress chaperones (Pan et al. 2015). In 2016, by transcriptome analysis, Potapova et al. identified cyclin D2 as a facilitator of adaptation to genome doubling in the presence of p53 in the human immortalized RPE-1 cells induced to tetraploidisation by knock-down of the essential regulator of cytokinesis, anillin (Potapova et al. 2016). This despite the presence of wild type p53 which is considered as a key inhibitor of cell proliferation and trigger of apoptosis of tetraploid cells. Crockford et al. confirmed in the human colorectal cancer cell line HCT116 and RPE-1 cell line that Cyclin D was specifically overexpressed in the early (not late passage) tetraploid clones and was sufficient to promote tolerance to spontaneous and pharmacologically induced tetraploidy (Crockford et al. 2017). These data indicate that that overexpression of cyclin D2 in diploid cells strongly potentiates the ability to proliferate with increased DNA content despite the presence of functional p53.

In 2017, Nakayama et al. observed that the v-Src oncogene weakens the tetraploidy checkpoint by inhibiting the nuclear exclusion of the transcription coactivator YAP, which is downstream of the Hippo pathway and its nuclear exclusion is critical in the tetraploidy checkpoint (Nakayama et al. 2017). In the same year, Do et al. observed that after cancer cell fusion, some fused cells avoid the apoptotic crisis partly owing to surviving, and continue to proliferate, a process that contributes to human cancer progressions (Do et al. 2017).

Recently, Bernhard et al. applied a RNAi mediated genome-wide screen in HCT116 after genome doubling induced by dihydrocytochalasin, an inhibitor of actomyosin ring and inducer of binucleated cells by blocking cytokinesis during telophase (Bernhard et al. 2022). They identified 140 genes whose depletion improved the survival of tetraploid cells and characterized in depth two of them: SPINT2, a general regulator of CdKN1, and USP28, affecting centrosome clustering through interaction with NuMA1.

In a recent paper, McKenney et al. explored the effects of ribotoxic stress and other stresses on cell cycle control in mammalian cells in culture (McKenney et al. 2024). They found that, stress-activated protein kinases led to the inhibition of the cyclin-dependent kinases CdK1, CdK4 and CdK6, thus allowing some cells to restart DNA replication without entering mitosis and to become polyploid. In this context, p53 function was not required, as cells deficient for the *p53* gene are still able to undergo this process. They concluded that CdK4/6 activity is required during G2 arrest to prevent stress-induced endoreplication and that persistent CdK inhibition by SAPKs may be an important source of whole genome doubling (WGD) in p53 deficient cells (McKenney et al. 2024).

The mechanisms favoring tolerance to unphysiological tetraploid cells and their proliferation deserve major attention to develop drugs targeting this critical and early step in disease development.

## Characteristics of the tetraploid cell

Tetraploidy implies per definition two identical genome complements, but what does it imply for the other constituents of the cell? The tetraploid cells that went through G2 (not after endoreduplication) are expected to have a doubled molecular equipment, including mitochondria and centrosomes. This amplification of centrosomes may lead to mitotic errors if the cell is not blocked at the tetraploidy G1 checkpoint and post-mitotic clustering of centrosomes is not achieved.

In multinucleated polyploid cells, it is assumed that nuclei progress in a synchronized way. However, Nano et al. observed in Drosophila neural stem cells that polyploid cell nuclei can proceed through cell cycle in an asynchronous manner (Nano et al. 2019). Delayed nuclei that are not yet competent to enter mitosis undergo DNA damage when exposed to the mitotic environment of neighboring cells.

Cell size control seems important. Hepatocytes were shown to remodel their metabolome as their size increased. A decrease of mitochondrial oxidative phosphorylation was observed with increasing cell size (Miettinen et al. 2014). However, the mechanisms leading to increase of cell size following polyploidisation are unknown (Cadart and Heald 2022).

The nucleus surface area-to-volume ratio decreases after genome duplication and mitotic slippage with possible consequences for chromatin organization. As far as histones are concerned, the data are scarce (for review, Ragusa and Vagnaretti 2023). During differentiation of trophoblasts, compared to undifferentiated (non-polyploid) counterparts, murine giant trophoblast cells generated by endoreduplication exhibited a reduction of expression of canonical histones but maintenance of high levels of histone variants H2A.X, H2A.Z ad H3.3 (Hayakawa et al. 2018). Upregulation of both canonical (H1, H2A, H2B and H3) and variant histones (H1.0, H2A.X and H3.3B) was identified in a mouse erythroleukemia cell line with a tetraploid phenotype acquired through resistance to differentiation (Fernández-Calleja et al. 2017). Several canonical histones (H2AC8, H2AC16, H2BC9, H2BC13, H3C7, H4C6) were found to be upregulated in polyploid giant cancer cells derived from MCF7 breast cancer lines (Antonelli et al. 2024) but rat hepatocytes treated with the polyploid-inducing carcinogen thioacetamide showed a downregulation of canonical H2AC1 (Mizukami et al. 2017).

Since it is well established that chromosomes occupy particular topologically associated domains (TADs) within the 3D-nucleus of diploid mammalian cells (for review Cremer and Cremer 2001; Duggal et al. 2014; McArthur and Capra 2021)), one may wonder what happens in a tetraploid nucleus. Organization of TADs in diploid versus tetraploid nuclei is well documented in plants, showing that chromatin organization, interactions between genomic regions and boundaries of TADs are different (for review Long et al. 2021; Verhage 2021)). Recent data in animal cells indicate that excessive DNA accumulation within one nucleus leads to loss of nuclear lamina interactions with lamina-associated domains, modifying the chromatin availability for transcription (for review (Anatskaya and Vinogradov 2022a, b)). Furthermore, recent studies indicate that genome duplications reorganize TADs within open A (interior nuclear space, transcriptionally active) and closed B (nuclear periphery, less transcriptionally active) chromatin compartments. This reorganization involves increased intra-TAD interactions, reconfiguration of chromatin loops, and oncogenic loss of chromatin segregation (Lambuta et al. 2023; Sas-Nowosielska and Bernas 2016; Wang et al. 2021). This leads also to the reorganization of the epigenome and additional changes of gene expression (for review (Recillas-Targa 2022)). Moreover, recent findings confirm that polyploidy drives epigenetic changes, including hypomethylation, histone modifications, and the replacement of canonical histones with non-canonical variants. These epigenetic alterations, along with the doubling of genetic material, are regarded as additional factors contributing to cellular plasticity, ultimately leading to phenotypic diversity (Anatskaya and Vinogradov 2022a, b; Feinberg and Levchenko 2023; Lambuta et al. 2023). For example, Gonzalez et al. found an association of the naturally occurring demethylation of SST1 pericentromeric repetitive elements and transposable elements LINE-1 with the onset of spontaneous tetraploidisation in human colorectal cancer (CRC) in culture and with TP53 mutations in primary CRCs cells (Gonzalez et al. 2021). Similar observations were described in ovarian cancer cells (Zeimet et al. 2011). It is therefore considered that polyploidy can regulate gene expression via chromatin opening, reawakening ancient evolutionary programs of embryonality (for review, (Erenpreisa et al. 2022)) and promote stress adaptation via epigenetic plasticity (for review, (Anatskaya and Vinogradov 2022a, b)).

As far as DNA replication is concerned, Gemble et al. made intriguing observations. They investigated the fate of RPE1 cells induced to become polyploid through mitotic slippage, cytokinesis failure, or endoreduplication. Their study revealed that freshly formed tetraploid cells, regardless of the induction mechanism, fail to accumulate sufficient DNA replication factors during the first G1 phase following whole-genome duplication. This deficiency leads to catastrophic DNA replication in the subsequent S phase, resulting in extensive karyotypic alterations (Gemble et al. 2022). Therefore, tetraploid cells might not be well equipped for DNA replication, just after tetraploidisation. If the tetraploid cells remain in mitotic arrest*, in casu* in physiological tetraploidy, no harmful consequences are expected but if they re-enter the cell cycle and are not eliminated at the G1-checkpoint, it may lead to mutations and diseases.

## Stress-induced tetraploidy: an adaptative metastable state

Unphysiological tetraploidization/polyploidisation is considered as one of the adaptive defense mechanisms against toxic stress above a given threshold (for review (Ghelli Luserna di Rorà et al. 2019; Sanz-Gomez et al. 2023)). It may protect from disease by “buffering” against genotoxic damages and reducing the chance of tumor suppressor loss (for review, (Matsumoto et al. 2021)). Moreover, several studies showed that WGD plays a suppressor role by inducing cell cycle arrest, cell senescence, apoptosis and even prompting cell differentiation (Decordier et al. 2008; Sanz-Gomez et al. 2023). Moreover, recently, tetraploidisation/polyploidisation was described as an adaptation against loss of heterozygosity (Archetti 2022).

As far as the molecular and cellular features of stress-induced unphysiological tetraploidy are concerned, they are expected to show similar imbalances as those described earlier (see Sect. 3) for physiological tetraploidy, with in addition modifications depending on the type of stress. These unique genetic and epigenetic characteristics do not confer a stable state to the cells, but a metastable state which may favor evolution towards disease.

The concept that stress-induced tetraploidy may correspond to a metastable state is not new. Polyploid cells were already described as metastable intermediates between diploidy and aneuploidy by Storchova and Kuffer (2008), Vitale et al. (2011a, b) and Hanselman and Welter (2016). More recently, we proposed a systemic approach of cancer development where tetraploidy is described as a metastable state towards malignant cell transformation. Detailed description of the mechanisms involved, including entropy changes, can be found in our review (Kirsch-Volders, et al. 2024). Whether tetraploidy may play a similar role in other diseases is unclear.

## Comparison of physiological and unphysiological stress-induced tetraploidy

Evolution of somatic cells to tetraploidy may be part of the programmed differentiation process (physiological) or resulting from abnormal stress (unphysiological). Why is tetraploidy/polyploidy favorable if it occurs in a programmed way in a specific tissue, but harmful if it develops in an uncontrolled way in another tissue? Comparison of both issues may help understanding the underlying processes. Table 1 summarizes the data described in the previous sections to allow a comparison for the type of inducer, the dose–effect relationships, the type of mechanisms involved, the tetraploidy checkpoint, S-phase, entropy state, fate, advantages and disadvantages.

**Table 1:** Comparison of physiological and unphysiological tetraploidy

Tetraploidy		
	Physiological	Unphysiological
Type of inducers		
Endogenous	e.g. muscular, thyroid hormone, fusogens	RONS and inflammations, Hypoxia
Exogenous	No	Genotoxicants, Neurotoxicants, Viruses
Dose effect		
	Hormone like	Thresholded?
Mechanisms		
Endoreduplication	Programmed	Induced
Mitotic slippage	No	Induced
Cytokinesis Inhibition	Programmed	Failure
Cell fusion	Programmed	Induced
Inducers of G1 Tetraploidy checkpoint	No	DNA replication stress, Abnormal centrosome dynamics
Fate		
Cycling	Low	Yes
Apoptosis/Senescence	Yes	Yes
3D reorganisation of chromatin		
Epigenetic changes	Yes	Yes
Transcript changes	Yes	Yes
Advantages		
Increased metabolic capacity	Yes	Yes / No
Protection against stressors		Adaptive response
Disadvantages		
Proliferation/Regenerative capacity	Low	+ cycling but aneuploidy, MN and inflammation
Tissue Repair	Low	Yes if cycling
Entropy status		
	Stable	Metastable

Physiological tetraploidy is a normal step during differentiation of many tissues. Unphysiological tetraploidy is induced as a stress response to either endogeneous or exogeneous stressors at a given threshold (for review (Ghelli Luserna di Rorà et al. 2019)). In addition to the mechanisms observed in programmed physiological tetraploidy, mitotic slippage occurs in unphysiological tetraploidy, leading to shortage of factors to fulfill accurate S-phase and DNA replication stress. The latter and abnormal centrosome dynamics are at the present time considered to be the key factors for the G1-tetraploidy checkpoint. It is therefore attractive to consider that mitotic slippage is linked to detrimental effects of unphysiological tetraploidy. The major disadvantage/risk of unphysiological tetraploidy occurs when tetraploid cells which underwent exposure to stressors and accumulated genetic changes, are not eliminated by apoptosis or immune surveillance, start cycling and leave their metastable state This leads to genetic instability, aneuploidy, inflammation and diseases. Although progresses were achieved, the key questions relevant to the mechanisms responsible for cellular tolerance to unphysiological tetraploidy and its consequence are not understood.

## Consequences of unphysiological tetraploidy

The consequences of this particular genetic, epigenetic, proteomic and metabolic state are multifactorial, follow different evolutions, leading to different outcomes, including diseases. In the following paragraphs, we will try to elucidate why and how stress-induced tetraploidy may lead to disease.

In a recent paper, we proposed a model showing how a metastable state may lead to cancer (Kirsch-Volders et al. 2024). In fact, a metastable state is of relatively long but finite lifetime. Under proliferative or genotoxic stress the previously accumulated or the accumulating gene mutations, the imbalance at DNA replication, epigenetic, transcription and proteome level at a given time destabilizes the unphysiological metastable tetraploid cells towards cell cycle entry, leading to aneuploidy, ploidy reversal with or without aneuploidy, pro-inflammatory micronuclei formation and neoplastic cell transformation (for review Ashcroft et al. 2015; Hanahan and Weinberg 2011; Kirsch-Volders et al. 2024)). However, it is not fully understood why and how a metastable stress-induced tetraploidy state transitions towards a specific new state, thereby leading to a specific disease, in particular especially for the diseases other than cancer.

### From tetraploidy to aneuploidy

Escape of tetraploid cells from the control checkpoints and re-entry in the cell cycle may lead to DNA damage, chromosome malsegregation and aneuploidy (for review (Decordier et al. 2008)). Many data demonstrated that cytokinetic failure-induced tetraploidy may develop into aneuploidy in rodents (Tanaka et al. 2015) and humans as well (for review, (Prasad et al. 2022)). Aneuploidisation following tetraploidisation is mainly observed in p53-deficient cells. It primarily arises from abnormal chromosome-chromosome and/or spindle-chromosome attachments in the context of excessive chromosome numbers, as well as from configurations involving extra centrosomes, which lead to multipolar spindles and subsequent mitotic errors (for review Potapova et al. 2016; Tanaka et al. 2018)).

### Fate of supernumerary centrioles

Figure 2 shows that tetraploid cells that arise via abnormal cell events, including cytokinesis failure, cell fusion, endoreduplication, and mitotic slippage present four centrioles. However, supernumerary centrioles are not always maintained in a cell population after genome duplication (for review, Bloomfield and Cimini 2023; Shin et al. 2021)). Galofré et al. observed that after tetraploidisation, cells reduce supernumerary centrosomes and that only a small number of cells retains extra centrosomes, potentially resulting in chromosome instability (Galofré et al. 2020). Baudoin, et al. compared p53-positive and p53-negative human cells in vitro, and found that populations of newly formed tetraploid cells quickly evolve to maintain a near-tetraploid chromosome number while losing the extra centrosomes acquired during tetraploidisation (Baudoin et al. 2020). This observation suggests a natural selection process where tetraploid cells that inherit a single centrosome during bipolar division—with asymmetric centrosome clustering—are favored for long-term survival. If centrosome clustering is the best-characterized mechanism to cope with extra-centrosomes, other mechanisms such as centriole inactivation, autophagy and clonal selection may also be involved (for review, Bloomfield and Cimini 2023; Shin et al. 2021)).

### MN formation

In addition to aneuploidisation, micronuclei (MN) may be induced if the chromosome(s) fails to attach to the spindle and lags behind in anaphase/telophase resulting in its exclusion from the main nuclei. (for review, (Fenech et al. 2011; Hashimoto and Todo 2013; Kirsch-Volders et al. 2020)). MN may either be reintegrated into the daughter cells at random or disappear after microphagy or membrane disruption leading to DNA leakage in the cytoplasm. In contrast, chromosome shattering within the MN results in massive chromosomal rearrangements following re-integration into the nucleus (for review, (Kirsch-Volders et al. 2020)).

### Inflammation

After polyploidization inflammation may be induced by different cellular responses. MN triggers inflammation through the release of cytosolic DNA, after micronuclear membrane rupture. Cytosolic DNA is detected by cGAS (cyclic GMP-AMP synthase), which, upon activation, triggers a signaling cascade that results in the production of type-1 interferons and pro-inflammatory cytokines (for review, (Kirsch-Volders and Fenech 2022)). Therefore, MN are not anymore considered as passive cytoplasmic passengers but are pro-inflammatory and pro-mutagenic entities contributing to disease development. Moreover, it may lead to a vicious cycle, since inflammation may induce MN and MN may in turn induce inflammation (Fenech et al. 2020; Kirsch-Volders et al. 2020).

Another trigger of inflammation in tetraploid cells is the presence of extra centrosomes. It was shown that extra centrosomes, arising during unscheduled polyploidization or aberrant centriole biogenesis, induce activation of NK-kappa B signaling and sterile inflammation. This signaling requires the NEMO-PIDDosome, a multi-protein complex composed of PIDD1, RIPK1, and NEMO/IKKgamma. Remarkably, the presence of supernumerary centrosomes suffices to induce a paracrine chemokine and cytokine profile, able to polarize macrophages into a proinflammatory phenotype (Garcia-Carpio et al. 2023).

### Deploidisation

**Fig. 3:**
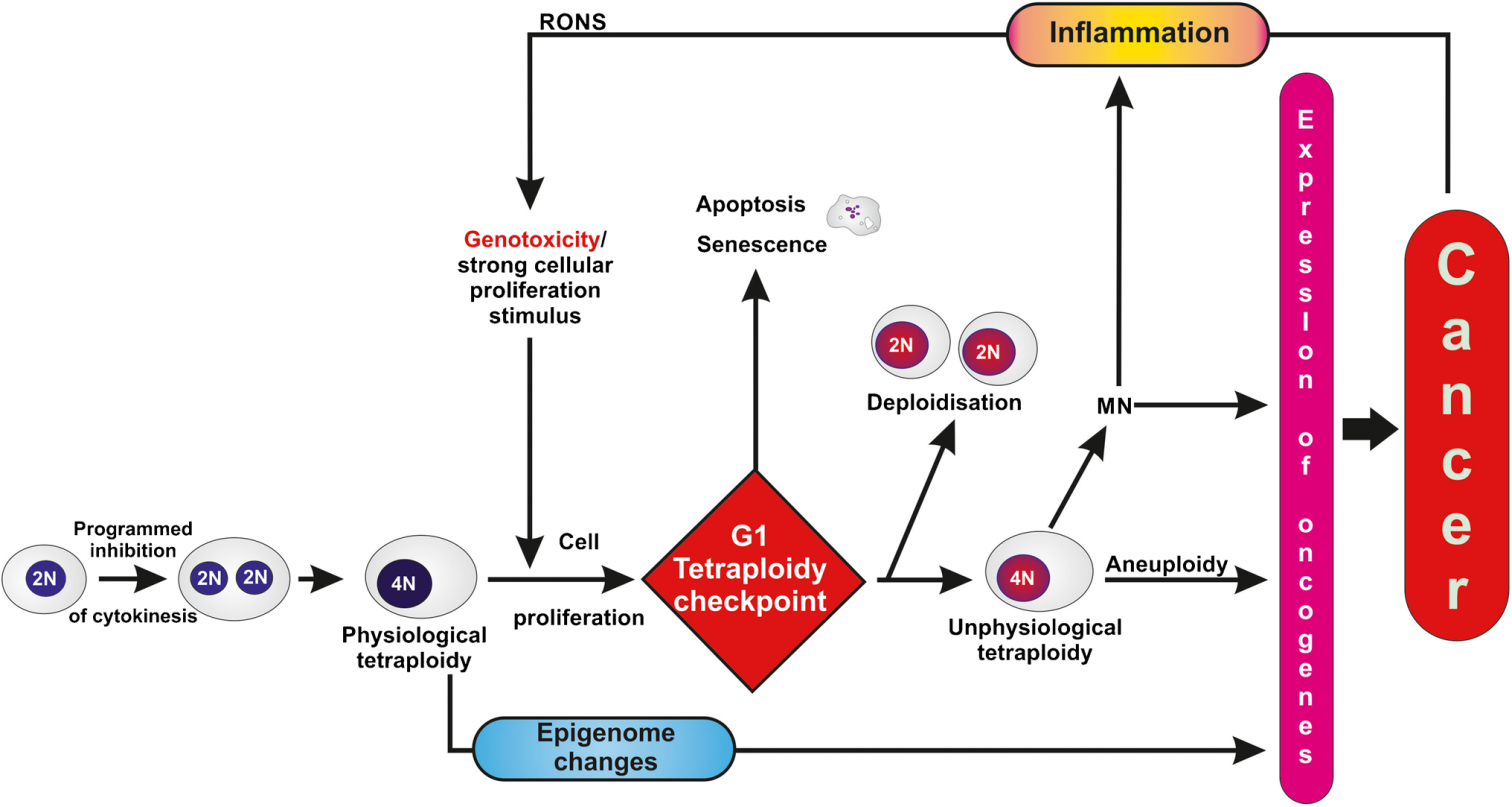
Schematic representation of the potential contribution of unphysiological tetraploidy to cancer. RONS—reactive oxygen and nitrogen species

It was shown in rodents, that following drug- and diet-induced liver injury, quiescent polyploid hepatocytes re-enter the cell cycle, undergo cytokinesis, reduce their polyploid state, and give rise to proliferating diploid cells. Similarly, partial hepatectomy stimulates cell proliferation, diploidization, and tissue regeneration (Fig. 3). These findings were already documented in the 1980s in studies investigating the role of polyploidy and cell proliferation in rat models of hepatocarcinogenesis (Castelain et al. 1989; Deleener et al. 1987; Haesen et al. 1988; Kirsch-Volders et al. 1988) (for review (Kirsch-Volders et al. 2024)). Combining molecular tools with histology, recent studies confirmed and complemented the earlier observations. As an example, Sladky et al. observed that cancer developed in low ploidy cells both in wild and deficient mice, the latter being characterized by a higher basal liver ploidy (Sladky et al. 2021). Their findings, along with those of other studies, suggest that proliferative polyploid hepatocytes can give rise to tumors through ploidy reduction. Furthermore, high ploidy appears to buffer against secondary genetic hits acquired later in life (for review, Lin et al. 2021; Matsumoto et al. 2021; Sladky et al. 2021; Yamazoe et al. 2020)). However, whether diploidisation occurs in tissues other than liver is not known and needs to be assessed.

Recently, the role of polyploidy reversal in the generation of pathological effects is attracting a lot of attention, with preventive and therapeutic objectives. Wu et al. and Zhao et al. reviewed the importance of polyploidization and depolyploidization in tissues and tumors, with a particular focus on polyaneuploid cancer cells (Wu et al. 2022; Zhao et al. 2024). They propose interesting models for the adaptive ploidy reversal mechanisms, such as multipolar mitosis, centrosome clustering, meiosis-like division, and amitosis.

### Stress-induced tetraploidy and diseases

As mentioned earlier tetraploidy/polyploidy is involved in several diseases, namely brain diseases, cardiovascular diseases, diabetes, wound healing and cancer (Anatskaya and Vinogradov 2022a, b). It is often not clear whether tetraploidy is a cause or consequence of the disease. Moreover, there exists a potential vicious cycle between tetraploidy and disease, since tetraploidy can induce diseases via genomic and epigenetic changes and that diseases may in turn induce tetraploidy through inflammation-dependent genetic stress and/or damage to the mitotic apparatus. The question here is whether stress-induced tetraploidy may be (co)-responsible of diseases: few data are available, except for cancer development.

#### Cancer

The link between polyploidy and cancer is well documented. First, tetraploid/polyploid cells are observed in most cancers (for review, Lambuta et al. 2023; Prasad et al. 2022)). Second, recent studies have indicated that polyploidy promotes cancer initiation, progression, metastasis, and drug resistance (for review, (Erenpreisa et al. 2015; Liu 2022; Loftus et al. 2022; Walen 2022)). Third, polyploidy-derived aneuploidy is considered to play a key role in carcinogenesis (for review, (Prasad et al. 2022)). Finally, a recent review describes within a systemic approach of cancer development how unphysiological tetraploidy induced by a genotoxic and/or strong proliferative stress is a metastable state leading to aneuploidisation, diploidisation, inflammation and malignant cell transformation (Kirsch-Volders et al. 2024) (Fig. 3).

In the following paragraphs, we bring together the data which may help identify the events triggering the evolution from physiological tetraploidy to unphysiological tetraploidy leading to neurodegenerative and cardiovascular disease. We pay particular attention to the well-known drivers of diseases triggered by stress-induced tetraploidy: chromosome instability, inflammation and deploidization (Kirsch-Volders et al. 2024).

#### Neurodegenerative diseases

As mentioned earlier (Sect. 2.1.4), recent studies with the nervous system discovered a link between cell-cycle reentry of neurons and several neurodegenerative conditions (for review Barrio-Alonso et al. 2018; Frade and Lopez-Sanchez 2017; Nandakumar et al. 2021)).

The cause of cell-cycle reentry in neurons may be cell loss or neurotoxicity, such as found in Alzheimer disease (Chao et al. 2020; Ippati et al. 2021). Neurotoxicants may be of different nature and origin (endogeneous or exogeneous) (Tywabi-Ngeva et al. 2022). For example, in Alzheimer disease, it is known that the amyloid-beta peptide, the major component of senile plaques in the brain, triggers cell-cycle re-entry in fully differentiated neurons and that this cell cycle re-entry protects from amyloid-beta toxicity and resultant cell death (Ippati et al. 2021).

As far as cell loss is considered, it is unclear whether cell cycle re-entry is the cause or consequence of cell loss. Cell cycle re-entry, if leading to unphysiological tetraploidy and/or aneuploidy, is known to induce apoptosis in neurons (for review (Yurov et al. 2019)). Neurodegeneration is considered as a consequence of those complex interactions but the genetic and epigenetic changes involved in neurodegeneration did not find a consensus yet. There are already several papers which describe an increase of tetraploidy in neurodegeneration (for review (Nandakumar et al. 2021)) but others consider that aneuploidy is the driving factor (for review (Yurov et al. 2019)).

**Fig. 4:**
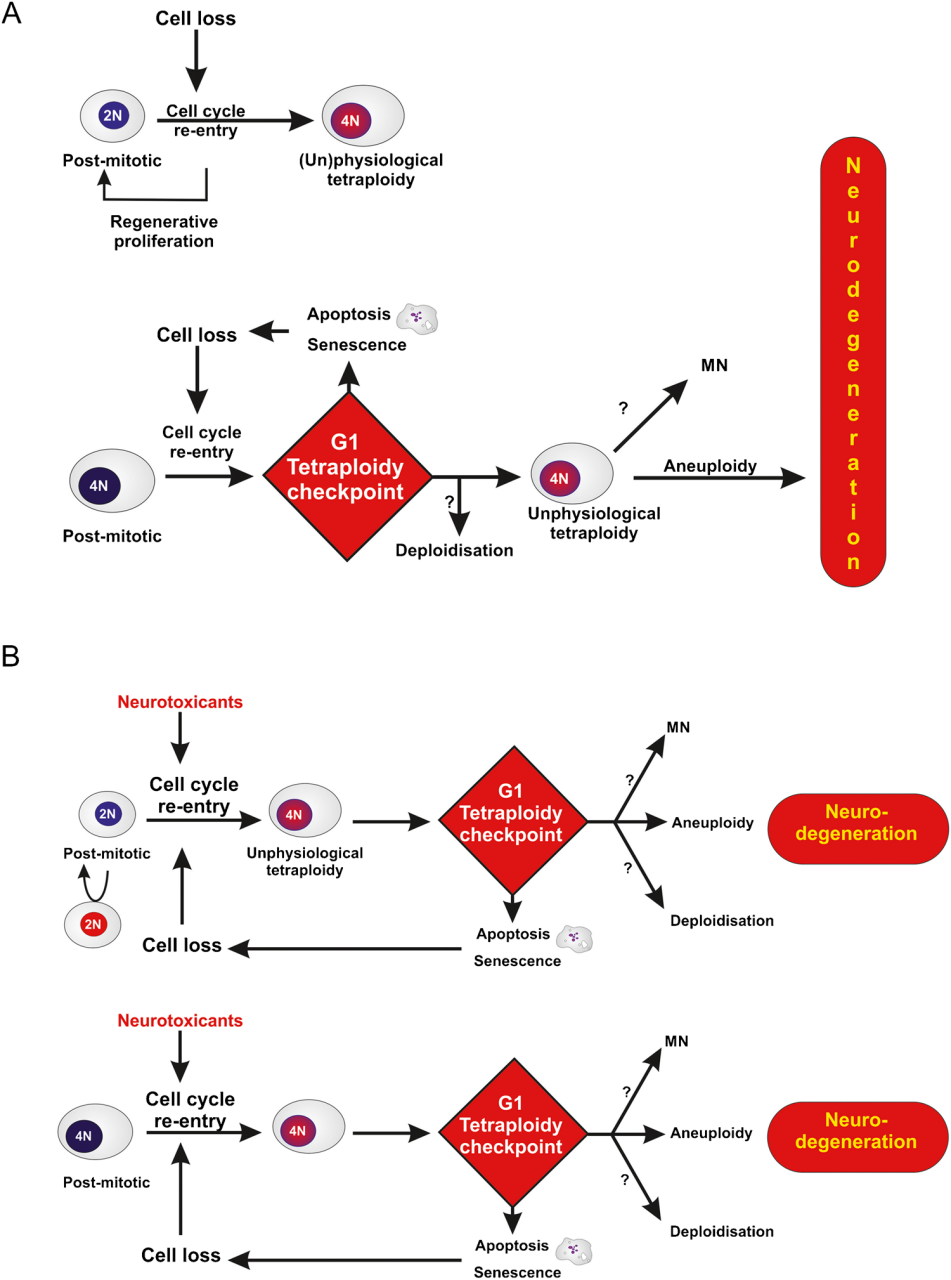
Schematic representation of the potential contribution of unphysiological tetraploidy to neurodegenerative diseases. The red color labeling tetraploid cells indicate that the cells underwent geno- or epigenomic toxic effects and therefore correspond to an unphysiological status

At the present time, it is thus not possible to propose a single model to describe the consequences of stress-induced cell cycle re-entry of neuron cells. In Fig. 4, hypothetical models are proposed, with cell loss as a cause in Fig. 4A and cell loss as a consequence in Fig. 4B.

Figure 4A compares the effect of chromosome loss on mature diploid or tetraploid post-mitotic neurons. In case of diploid cells, cell loss is considered to lead either to re-entry in the cell cycle followed by proliferation of normal diploid cells (or to the formation of a tetraploid cell with protective function, if the proliferative stress is too strong). In case of tetraploid cells, re-entry into the cell cycle may trigger MN induction, aneuploidy and/or possibly deploidisation, leading to neurodegeneration. These mechanisms are well known for cancer development but not yet demonstrated for neurodegeneration (for review, (Kirsch-Volders et al. 2024)). In addition, apoptosis followed by a second cycle of chromosome loss may be induced by the tetraploid status.

Figure 4B considers chromosome loss as a consequence of neurotoxicants and compares the effect of neurotoxicants on mature diploid or tetraploid post-mitotic neurons. In case of diploid cells, the neurotoxicants induce re-entry in the cell cycle, proliferation of diploid cells or formation of tetraploid cells, both presenting genetic and/or epigenetic modifications and pro-neurodegenerative effects. In case of tetraploid cells, if the neurons escape the G1-tetraploid checkpoint, one expects induction of MN, aneuploidisation and possibly deploidisation. In addition, the neurotoxic effects may induce apoptosis and cell loss in both diploid and tetraploid cells.

These models are not intending to cover all possible pro-neurodegenerative processes but underline the contribution of unphysiological tetraploidy which was often overlooked. For clarity reasons, they do not include the involvement of higher polyploid levels. The mechanisms described show some similarities with induction of cancer where a strong cell proliferation stimulus or genotoxic stress modifies the balance of the metastable stress-induced tetraploidy towards aneuploidy induction, inflammation and neoplastic cell transformation (Fig. 3) (for review, (Kirsch-Volders et al. 2024)). This provides some support for the contribution of chromosome instability to brain neurodegeneration (for review, (Yurov et al. 2019)). However, a key difference between carcinogenesis and neurodegeneration results from the capacity of neurons to activate apoptosis even in pathological contexts and cancer cells do not (for review, (Hollville et al. 2019)). To progress further a better understanding of the pathophysiological effects of neuronal tetraploidy is necessary. Tetraploidy-triggered neurodegeneration might be linked to changes of cell size, alteration of synaptic transmission due to a hypothetical changes of dendritic and axonal size or aging (for review, (Frade and Lopez-Sanchez 2017)). Interestingly, Kundra et al. demonstrated that protein homeostasis of a metastable subproteome is associated with Alzheimer disease: a too high concentration of amyloid-beta peptide (amyloid plaques) and the tau protein (neurofibrillary tangles) resulting from inadequate protein trafficking and clearance leads to its aggregation and neurodegeneration (Kundra et al. 2017). One may hypothesize that in tetraploid cells, an increased synthesis of this protein and/or its lower solubility in the changed surface area-to-volume ratio of the tetraploid cell may contribute to its abnormal concentration.

#### Cardiovascular diseases

**Fig. 5:**
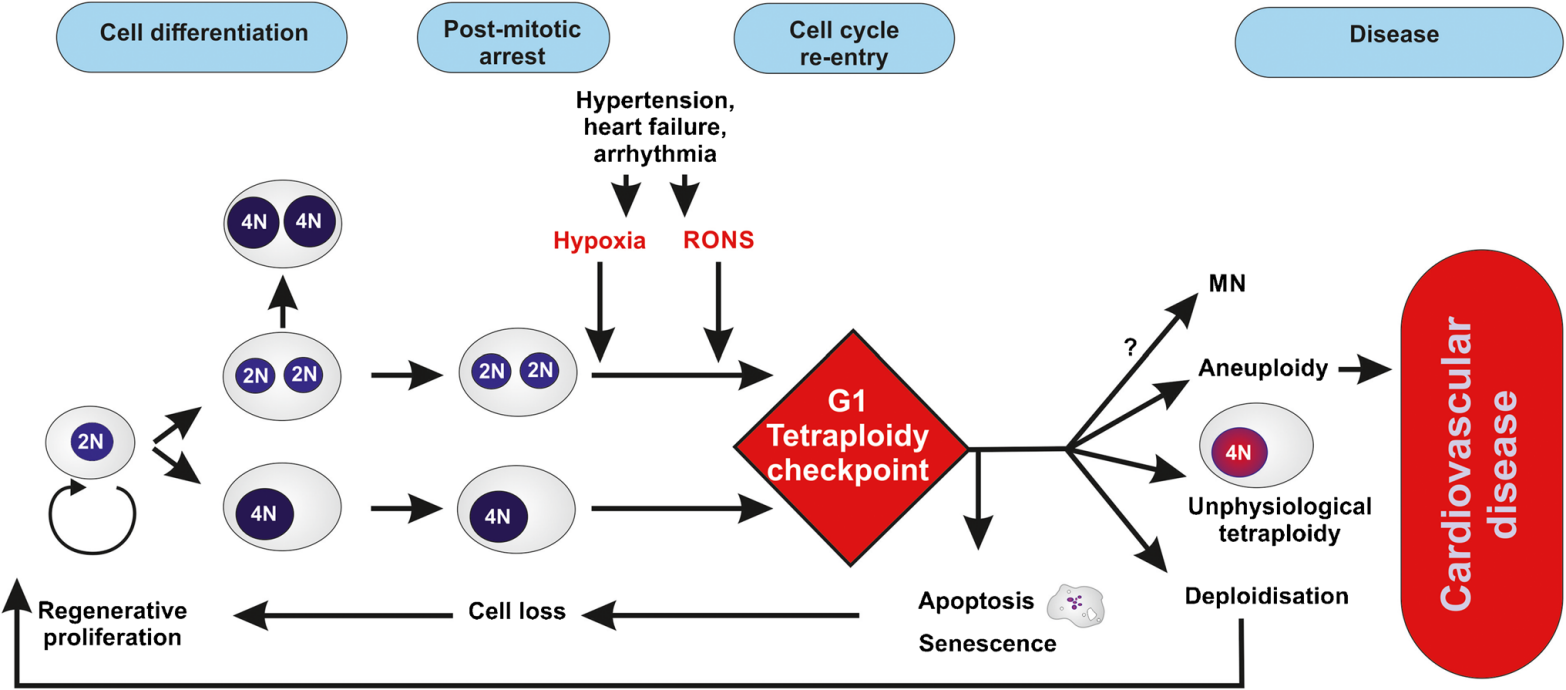
Schematic representation of the potential contribution of unphysiological tetraploidy to cardio-vascular diseases. RONS—reactive oxygen and nitrogen species

As far as mature cardiomyocytes are concerned, the probable triggers for stress-induced tetraploidy are hypertension leading to hypertrophy, ischemic and non-ischemic heart failure and arrhythmia (for review Buddell et al. 2024; Derks and Bergmann 2020)). Interestingly, during differentiation of cardiomyocytes changes in oxidative balance, including hypoxia, trigger physiological polyploidization ((Jiang et al. 2020); for review (Derks and Bergmann 2020; Elia et al. 2023)). Since oxidative stress accompanies heart failure, its role in induction of unphysiological tetraploidy is highly probable (Derks and Bergmann 2020; Elia et al. 2023). Based on our knowledge concerning hepatocytes and neurons, we propose a hypothetical model for the fate of stress-induced tetraploid mononucleated and binucleated cardiomyocytes (Fig. 5). However, currently no data are available on the induction of chromosome instability, formation of micronuclei leading to inflammation or deploidisation after stress-induced tetraploidisation in cardiomyocytes. It is possible that MN have not been reported in cardiomyocytes, due to the rare occurrence of mitotic events (for review, (Kirillova et al. 2021)). Moreover, in human cardiomyocytes after loss of cardiac cells following injuries and heart disorders, cell regeneration by reentry in the cell cycle occurs only in diploid cells and was not observed in tetraploid cells. Polyploidy might therefore limit cardiomyocytes proliferation and regeneration following injury (for review, (Elia et al. 2023)). However, in regenerative organisms, cardiomyocytes revert from their terminally differentiated state into less mature state (i.e., dedifferentiation) to allow proliferation and regeneration to occur, suggesting that deploidisation may occur under stress ((Beisaw and Wu 2024; Kirillova et al. 2021)).

When comparing the data available on the contribution of stress-induced tetraploidy to cancer versus neurodegenerative and cardiovascular diseases, some differences and similarities appear: i) apoptotic capacity is maintained in neurons and cardiomyocytes but not in cancer cells; ii) deploidisation allowing reversal from tetraploidy to diploidy and favoring regeneration is until now only found in cancer cells; iii) aneuploidy is found in cancer cells and neurodegenerative neurons but not observed yet in cardiomyocytes; iv) the role of MN towards inflammation is described only in cancer development.

In conclusion, the role of stress-induced tetraploidy in the onset of diseases other than cancer is highly probable but unclear. The mechanisms leading to polyploidization show similarities but also differences. Moreover, the mechanisms responsible for the exit from the metastable stress-induced tetraploid cell to the disease-prone state are not understood; therefore, genetic, epigenetic, metabolic and proteomic data are needed in a cell-by-cell approach along the emergence of the disease starting from the tetraploid cells.

## Nutritional and pharmacological prevention and/or elimination of unphysiological tetraploidy

### Nutritional prevention

Nutritional prevention should be a priority to avoid induction of tetraploidy. Knowledge about dietary factors that may decrease risk of polyploidy and the use of this knowledge for prevention is sparse, but some recent studies suggest it may be plausible.

**Fig. 6:**
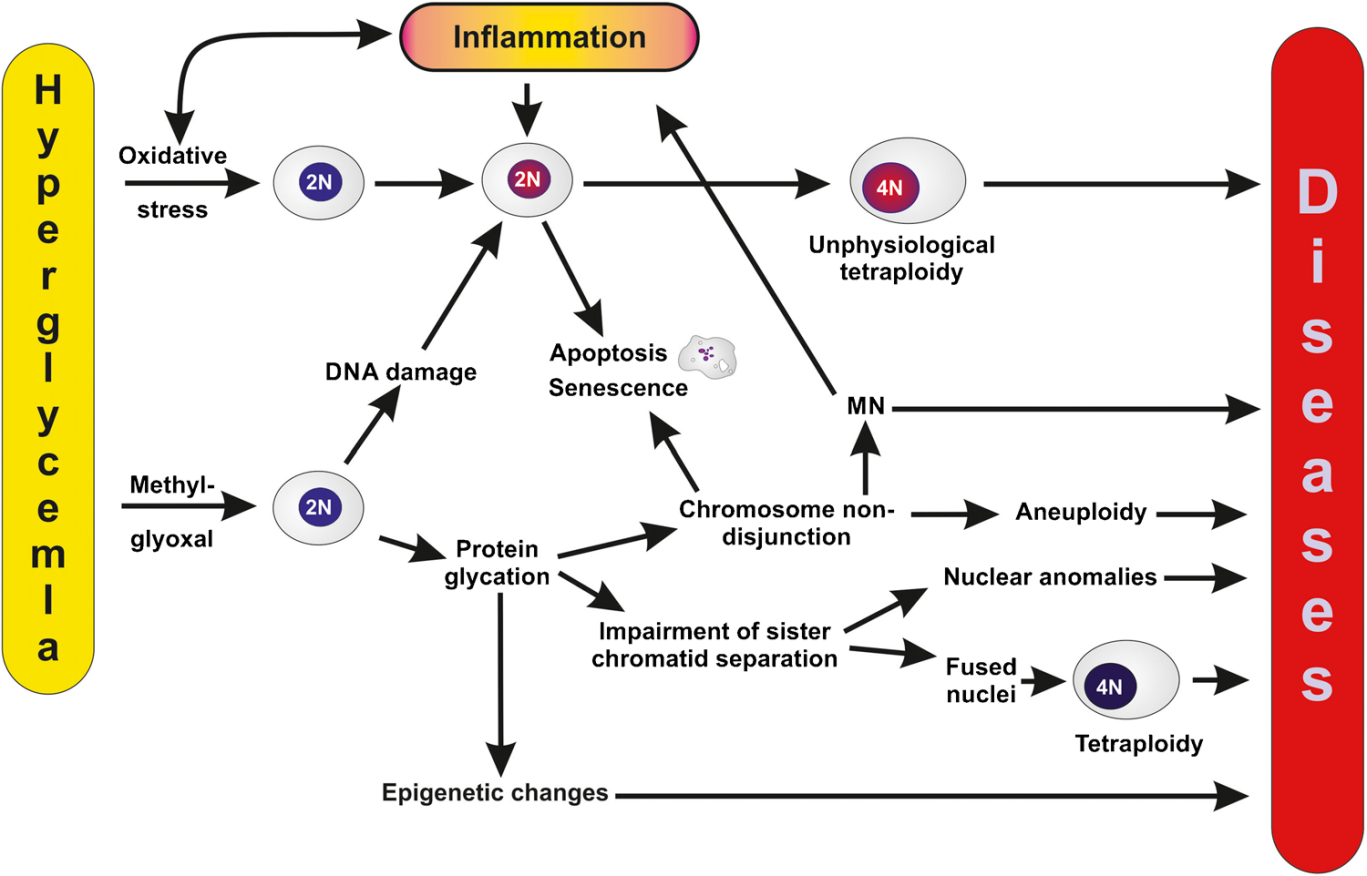
Schematic representation of the potential contribution of hyperglycemia to genomic instability and diseases

For example, folate deficiency can cause telomere loss and dysfunction possibly by hypomethylation of sub-telomeric DNA and histones (Vera et al. 2008) which may lead to failure of chromatid separation at telomeres and generation of fused nuclei caused by multiple nucleoplasmic bridges and the formation of tetraploid cells by mitotic slippage (Donnellan et al. 2021). Furthermore, recent in vitro studies showed that methylglyoxal (MGO), a reactive dicarbonyl by-product of glycolysis that is elevated in type 2 diabetes, causes mitotic dysfunction and complex nuclear anomalies, suggestive of mitotic slippage and aneuploidy, by preventing sister chromatid separation (Donnellan et al. 2021; Donnellan et al. 2022); these cellular disorders and other pathologies induced by MGO suggest that dietary intervention aimed at reducing MGO concentration in the body may be a relevant anti-aneugenic and anti-ageing strategy (Schalkwijk and Stehouwer 2020). Furthermore, it is becoming increasingly ´evident that chromosomal histone modifications are altered during mitosis in chromosomal regions that are critical for chromosome segregation such as regions involved in cohesion, centromere function, kinetochore engagement and telomere maintenance (Schmitz et al. 2020). Given that post-translational modifications of histones are epigenetic marks that are modifiable by nutritional factors it is not implausible that specific micronutrient deficiencies (such as deficiencies of folate and NAD) may also exert polyploidogenic effects by affecting the degree of histone modifications (Fig. 6) (Fan et al. 2015).

Another approach to reducing polyploid cells or their senescent versions within the body is to identify phytochemicals that either selectively slow down their regenerative potential relative to normal diploid cells or to induce their death. For example, Lissa et al. identified resveratrol and salicylate as compounds that kill tetraploid cells more efficiently than their parental diploid counterparts both in in vitro and in vivo models (Lissa et al. 2014).

### Pharmacological prevention

Pharmacological prevention from induction of unphysiological tetraploidy consists in the avoidance of the stressors responsible for un-programmed endoreduplication, cytokinesis failure, mitotic slippage or un-programmed cell fusion (see Sects. 2.1 and 2.2), stimulation of the checkpoints controlling the accuracy of the mitotic machinery or potentialisation of apoptosis (for review (Kirsch-Volders and Fenech 2023)). As an example, in tumors, combination therapies are developed to potentiate apoptosis of mitotically-inhibited cells before they progress to tetraploidization (Havas et al. 2016; Motwani et al. 2000; Sedlacek 2001) (for review (Zhang et al. 2020a, b)).

Once an unphysiological tetraploid cell emerges, the first step aiming at its elimination depends on the activity of the G1-tetraploid checkpoint. However, some tetraploid cells escape this surveillance and become the target of therapeutic elimination strategies (see Sect. 2.4).

Recently, Zhang et al. nicely reviewed the emerging strategies to attack existing polyploid cancer cells (Zhang et al. 2020a, b). A first strategy is similar to that applied for avoidance of tetraploidization: stimulating apoptosis with or without combination treatment. For example, i*n vitro* studies showed that inhibition of Chk1 kills tetraploid tumor cells through a p53-dependent pathway (Vitale et al. 2007).

Another strategy uses the higher sensitivity of tetraploid cancer cells as compared to diploid cancer cells. This selective killing may be based on gene dosage, such as 8-azaguanine metabolism (Choudhary et al. 2016) or disruption of molecular interactions (for review, (Zhang et al. 2020a, b)). On the other hand, in a recent review, tetraploid cancer cells were described as less sensitive than diploid cancer cells for some targets. They listed all the studies that targeted preferentially tetraploid tumor cells focusing on mitosis machinery, essentially the spindle apparatus, in particular the centrosome apparatus (Eg5, HSET, PLK1) and the spindle assembly checkpoint pathways (MPS1, MAD2 and BUBR1, Aurora B, CHK1). The published studies demonstrated a preferential effect on tetraploid cancer cells compared to diploid cancer cells, when targeting the mitotic apparatus (Jemaà 2021). For example, in vitro, tetraploid cancer cells are more susceptible than diploid cancer cells to anti-mitotic stabilizer paclitaxel or inhibitors of a mitotic kinase MPS1, Plk1, or a mitotic kinesin motor protein Kifl18A (for review (Yoshizawa et al. 2023)). It was also found that tetraploid cancer cells are significantly more susceptible to inhibitors of a mitotic kinesin CENP-E than diploids and that CENP-E inhibitor had superior generality to paclitaxel in its tetraploidy selectivity across a broader spectrum of cell lines (Yoshizawa et al. 2023). This indicates that there is no general rule for the molecular and cellular characteristics of (near)-tetraploid cells: all depends on their genetic background, the target and the stressor, rendering therapy extremely challenging.

A promising research line to prevent the development of polyploid cells focusses on the high energy cost for maintenance of polyploidy as compared to diploidy. It was shown that co-treatment with mTOR (master regulator of energy) inhibitors increased the anti-cancer effect of Aurora kinase B inhibitors in vitro in myeloid leukemia cell lines (Liu et al. 2013). In the same context, resveratrol or salicylate (active product of aspirin) which activate the 5’ AMP-activate protein kinase (AMPK), a direct upstream inhibitor of mTOR, reduced the formation of tetraploid or higher-order polyploid cells in human colon carcinoma cell lines or primary mouse epithelial cells lacking p53 in the presence of antimitotic agents (Lissa et al. 2014). Moreover, oral treatment with either resveratrol or aspirin repressed the accumulation of tetraploid intestinal epithelial cells in the Apc ^Min/+^ mouse model of colon cancer (Lissa et al. 2014).

Although very promising, these strategies are not easily applicable because they may eliminate diploid and physiological tetraploid cells. The ultimate objective is to eliminate specifically unphysiological tetraploid cells and to spare diploid and physiological tetraploid cells present in normal tissues. Büttner et al. observed in vitro that depletion of endonuclease G kills tetraploid but not diploid clones of the human HCT 116 colon carcinoma cell line during exponential growth (Buttner et al. 2007). Interestingly, Quinton, et al. used sequencing data from circa 10 000 primary human cancer samples and essentially circa 600 cancer cell lines and showed that WGD gives rise to common genetic traits that are accompanied by unique vulnerabilities: WGD^+c^ cancer cells are more dependent on spindle assembly checkpoint signaling, DNA replication factors and proteasome function than WGD^−^cells (Quinton et al. 2021).^.^ They also identified KIF18A, encoding for a mitotic kinesin, as being specifically required for the viability of WGD^+^ cells. This suggest that exploiting the specific vulnerabilities of tetraploid tumor cells might be a valuable strategy to specifically target WGD^+^ cancer cells while sparing the normal, non-transformed WGD- cells that comprise human tissue.

### Immunosurveillance

As compared to the mechanisms described above, immunosurveillance against tetraploidisation may be the most specific strategy (see Sect. 2.3.2). An interesting perspective might be the use of calreticulin as an immunological target (Senovilla et al. 2012). The recent successes obtained with immunotherapies in combating cancer provide good hope that specific immunotherapies against unphysiological tetraploidy will be available in the future.

In conclusion, from the different strategies described briefly above, more research on translation into practice of nutritional prevention is highly recommended. Elimination of tetraploid cells and in particular unphysiological tetraploid cells remains unsolved yet. At the present time, combination of different strategies remains the better option.

## Knowledge gaps and future perspectives

Although the importance of tetraploidisation/polyploidisation in evolution, development, differentiation, tissue function and repair, defense against stress and its role in diseases is now well established, the mechanisms responsible for the transition from physiological to unphysiological tetraploidy, the tolerance to tetraploidisation in unphysiological tetraploidy and the transition from the metastable stress-induced tetraploidy towards disease are not understood.

We proposed some hypothetic models for the contribution of tetraploidy towards neurodegenerative (Fig. 4) and cardiovascular (Fig. 5) diseases in comparison with its role in cancer development (Fig. 3). However, several difficulties/shortcomings remain and render the explanations and conclusions difficult:

- Most of the data on unphysiological tetraploidy, with few exceptions in recent cancers studies, are obtained in vitro. More in vivo data are needed. Concerning diseases other than cancer, in vitro*/*in vivo models should be further developed and validated. This issue is particularly difficult for neurodegenerative diseases. Use of stem cells and organoids provides interesting opportunities and would be helpful for treatment of cardiovascular diseases, in particular. An interesting example was provided recently by Darmasaputra et al. on human hepatocyte organoids showing the evolutionary conservation of WNT signaling along with E2F7 and E2F8 in the regulation of endomitosis (Darmasaputra et al. 2024).

- Identification of the key controllers of tetraploidy checkpoints is complex. For instance, p53, which is considered as essential, can be induced by so many different stressors, rendering the causal link between a given stressor and p53 difficult.

- Most of data are not specific enough and heterogeneous, since tetraploidy can be induced by different mechanisms and/or different stressors, leading either to binucleated polyploid or mononucleated polyploid cells.

- Dose–effect relationships to assess how the level of stress direct the fate of the cells, are missing. It is crucial to know whether the decision of the cell to promote mitotic slippage, apoptosis, senescence or further cycling is related to a threshold.

- The transition from the metastable tetraploidy state to aneuploidy and disease is complex but its importance to prevent and treat disease is evident. To unravel the mechanisms responsible for the exit from the metastable tetraploid cell to the disease-prone state, genetic, epigenetic, metabolic and proteomic data are needed in a cell-by-cell approach along the emergence of the disease starting from the tetraploid cells.

- Addressing tetraploidy in cell lines requires well characterized tetraploid cell populations, as to their origin and induction method (Kirsch-Volders et al. 2003). Recently Shenk and Ganem developed a new method to generate and purify well defined tetraploid RPE-1 cells, by using the fluorescent, ubiquitin-based, cell cycle indicator (FUCCI) system (Bernhard et al. 2023; Shenk and Ganem 2016). This is a major progress into the good direction but the better solution would be to start from non-cancer primary cells, to avoid bias of acquired genome instability.

Combining cellular, cytogenetic, genetic and epigenetic data in a cell-by-cell approach would facilitate the understanding of the contribution of unphysiological tetraploidization to the onset of diseases.

New approaches should also be explored, such as 3D chromatin organization and entropy levels. The recent data demonstrated that tetraploidy, and chromosomal aberrations, modify the relative position of TADS and their interactions, leading to changes of epigenetic organization and transcription. Cellular plasticity due to tetraploidization should therefore be considered to result from both genetic (whole genome doubling providing gene amplification and protection) and epigenetic (resulting from 3D-reorganisation of chromatin) changes.

Genetic and epigenetic changes are also studied in terms of energy and entropy. It might be challenging and interesting to situate tetraploidisation in the frame of developmental dynamics of multicellular organisms, a process that takes place in a multi-stable system in which each attractor state represents a cell type, and attractor transitions correspond to cell differentiation paths.

In conclusion, tetraploidization in somatic cells is a phenomenon, conserved over evolution, providing genetic and epigenetic plasticity for adequate differentiation and opportunistic defense but deleterious in case of deficiency of the G1 tetraploidy checkpoint, allowing uncontrolled proliferation. Avoiding and elimination of unphysiological tetraploidy will require adequate prevention and further research.

## Data Availability

No datasets were generated or analysed during the current study.

## Abbreviations

2N: Diploid
4N: Tetraploid
AMPK: 5’ AMP-activate protein kinase
APC: Adenomatous polyposis coli
APC/C: Anaphase-promoting complex/cyclosome
CdK: Cyclin-dependent kinase
CRC: Human colorectal cancer
FUCCI: Fluorescent Ubiquitination-based Cell Cycle Indicator
HPV: Human papillomavirus
MCC: Mitotic checkpoint complex
MGO: Methylglyoxal
miR: MicroRNA
mTOR: Mammalian target of rapamycin
PLK-4: Polo-like kinase 4
RONS: Reactive oxygen and nitrogen species
SAC: Spindle assembly checkpoint
TADs: Topologically associated domains
WGD: Whole genome doubling
